# Nanoscale Terahertz Monitoring on Multiphase Dynamic Assembly of Nanoparticles under Aqueous Environment

**DOI:** 10.1002/advs.202004826

**Published:** 2021-03-24

**Authors:** Eui‐Sang Yu, Sang‐Hun Lee, Geon Lee, Q‐Han Park, Aram J. Chung, Minah Seo, Yong‐Sang Ryu

**Affiliations:** ^1^ Sensor System Research Centre Korea Institute of Science and Technology Seoul 02792 Republic of Korea; ^2^ Department of Optical Engineering Kumoh National Institute of Technology Gumi 39253 Republic of Korea; ^3^ Department of Physics Korea University Seoul 02841 Republic of Korea; ^4^ School of Biomedical Engineering Korea University Seoul 02841 Republic of Korea; ^5^ KU‐KIST Graduate School of Converging Science and Technology Korea University Seoul 02481 Republic of Korea

**Keywords:** nanoparticles, nanophotonics and plasmonics, nanoscale electrical tweezers, optical biosensors, terahertz optics

## Abstract

Probing the kinetic evolution of nanoparticle (NP) growth in liquids is essential for understanding complex nano‐phases and their corresponding functions. Terahertz (THz) sensing, an emerging technology for next‐generation laser photonics, has been developed with unique photonic features, including label‐free, non‐destructive, and molecular‐specific spectral characteristics. Recently, metasurface‐based sensing platforms have helped trace biomolecules by overcoming low THz absorption cross‐sectional limits. However, the direct probing of THz signals in aqueous environments remains difficult. Here, the authors report that vertically aligned nanogap‐hybridized metasurfaces can efficiently trap traveling NPs in the sensing region, thus enabling us to monitor the real‐time kinetic evolution of NP assemblies in liquids. The THz photonics approach, together with an electric tweezing technique via spatially matching optical hotspots to particle trapping sites with a nanoscale spatial resolution, is highly promising for underwater THz analysis, forging a route toward unraveling the physicochemical events of nature within an ultra‐broadband wavelength regime.

## Graphical abstract

Probing evolution process of nanomaterials in liquids is crucial for understanding mechanisms of physicochemical/biological events. Although terahertz (THz) regime provides intra/intermolecular information, large absorptive loss of THz energy underwater has limited practical biosensing applications. Here, the authors present bi‐layered architecture of nanoslot arrays to match electric tweezer with photonic hotspots, which allows real‐time THz monitoring of dimensional assembling of nanoparticle.

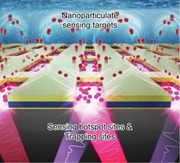

## 1 Introduction

Monitoring the kinetic processes of nanoparticle (NP) assembly in liquids is crucial for understanding the physicochemical reactions associated with nanophase evolution.^[^ ^1^ ^]^ A variety of optical techniques with various wavelengths have been employed to investigate underwater NP behaviors via seeing‐through technologies.^[^ ^2^ ^]^ In contrast with classical seeing‐through methods, including mid‐infrared and Raman spectroscopy,^[^ ^3^ ^]^ the low photon energy (0.4–40 meV) of terahertz (THz) light matches the intermolecular energy levels of molecules,^[^ ^4^ ^]^ providing unique molecular fingerprint information such as that regarding intra‐/intermolecular vibration modes at the broadband regime (0.1–10 THz).^[^ ^5^ ^]^ Therefore, THz spectroscopy is expected to be a next‐generation wavelength regime as an emerging frontier for investigating molecular phases and the corresponding functions.^[^ ^6^ ^]^ However, the small THz absorption cross‐section caused by the scale mismatch between the THz wavelength (30 µm–3 mm) and small sized molecular assemblies (< 100 nm) hinders the ultrasensitive THz detection of particulate molecular objects at low concentrations. Recently, metasurfaces and nanoantenna‐based surface‐enhanced spectroscopies^[^ ^7^ ^]^ have improved molecular detectability via enhanced THz absorption cross‐section at the nanostructured optical hotspots where the THz signal is maximized.^[^ ^8^ ^]^ Although metasurface‐assisted methodologies create opportunities for THz molecular detection in a highly sensitive and selective manner, significant absorptive loss of THz light by molecule‐surrounded water remains a long‐standing hurdle.^[^ ^9^ ^]^ Aside from avoiding signal loss via water absorption, biomolecules suspended underwater should be positioned near optical hotspots in sufficient amounts to yield noticeable THz signal changes over the optical limits of detection. As biochemical phenomena eventualize underwater, the development of a clever strategy to monitor and detect THz signals during the dynamic evolution of NPs from single units to volumetric assembly is highly desirable.

**Figure 1:**
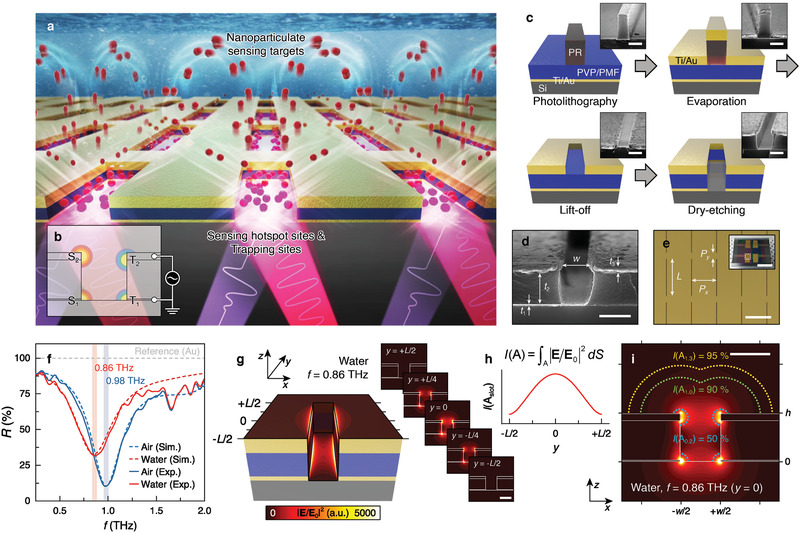
Nanoslot THz‐DEP sensor for electric NP capture into optical hotspots. a) Conceptual illustration of THz optical detection accompanied by electric NP capture into nanoslots. b) Schematic illustration of optical hotspots (S_1_ and S_2_) matching with trapping sites (T_1_ and T_2_). c) Fabrication process (insets show side‐view SEM images, scale bars = 500 nm). d) Side‐view of single nanoslot. Scale bar = 500 nm. e) Micrograph of nanoslot array. Scale bar = 40 µm. (inset shows photograph of entire device, scale bar = 1 cm). f) Calculated (dotted line) and measured (solid line) far‐field THz reflectance spectra under air (blue) and water (red) environments. g–i) Calculated electric near‐field enhancement (under water, *f* = 0.86 THz) over nanoslot g) insets show series of side‐view distributions), with near‐field intensity inside nanoslot along longitudinal direction h), and cross‐section at center of nanoslot exhibiting field intensity coverage for different distances from S_1_ and S_2_ i). Scale bars = 500 nm.

Herein, we report a novel type of large‐area electric‐photonic tweezer that actively controls the suspended NPs within a bulky water reservoir and captures them into predefined nanovolumes (**Figure** **1a**). By utilizing vertically‐aligned nanoantennas as electrodes, convection‐assisted delivery and low‐voltage capture localize extremely diluted NPs into electric trapping sites (T_1_ and T_2_ in Figure 1b). Simultaneously, adopting nanoantennas as NP detectors, the real‐time probing of captured NPs in an aqueous environment is examined via colossal THz electric field (*E*‐field) enhancement within optical hotspot sites (S_1_ and S_2_; Figure 1b). The electric‐photonic tweezer, enabling displacement‐sensitive NP detection, helps to understand the kinetic evolution of NP assembly in multi‐phase steps while being accumulated into the nanovolume.

## 2 Results

### 2.1 Design of Nanoslot Antenna Array and Optical Characterization

A nanoslot array was chosen for local field enhancement by its strong antenna resonance at the frequency of interest within the THz range.^[^ ^10^ ^]^ The resonance frequency (*f*_res_) of the nanoslot is theoretically given by $f_{\mathrm{res}}=c_{0}/L\sqrt{2({n_{\mathrm{sub}}}^{2}+{n_{\mathrm{med}}}^{2})}$, where *c*_0_, *n*_sub_, and *n*_med_ denote the speed of light and the respective refractive indices of the substrate and medium.^[^ ^11^ ^]^ Slot length (*L*) was designed to be 60 µm for the theoretical resonance conditions of *f*_res_ = 0.99 and 0.86 THz under ambient and aqueous conditions, respectively. To suppress the Rayleigh minima,^[^ ^12^ ^]^ nanoslots with widths (*w*) of 500 nm were arranged with transversal‐ (*P_x_*) and longitudinal (*P_y_*) periodic separations of 40 and 10 µm, respectively. Uniquely designed double nanoslot layers, building both a vertical nanogap for electrical tweezers and horizontal nanoslots for THz field enhancement, were prepared over large‐area (2 × 2 mm^2^) chips. The thicknesses of the bottom‐ (*d*_1_), insulator‐ (*d*_2_), and top layers (*d*_3_) were 25, 500, and 100 nm (thicker than the skin depth of 85 nm at 1.0 THz^[^ ^13^ ^]^), respectively (Figure 1c, Experimental Section, and Note S1, Supporting Information). The side‐view scanning electron microscopy (SEM) images and micrographs demonstrate large‐area structural fidelity with excellent production yields, which is an essential prerequisite for signal acquisition that is reliable enough to cover the THz beam spot with a diameter of 1 mm (Figure 1d,e).

To validate the design and fabrication of the nanoslot array, far‐field THz reflectance (*R*) was simulated (Note S2, Supporting Information) and experimentally corroborated using THz time‐domain spectroscopy in reflection mode (THz TDS, Experimental Section). Both the simulation and experimental results present nanoslot reflectance resonance (*R*_res_) at *f*_res_ = 0.98 and 0.86 THz in air and water environments, respectively (Figure 1f). The excellent correspondence between the simulation and measurement substantiates that a nanoslot array was uniformly fabricated over a large area. Under aqueous resonance conditions (*f*_res_ = 0.86 THz), the local THz field distribution exhibits a strongly confined near‐field region at the sharp metallic edges of the nanoslot (S_1_ and S_2_, Figure 1g). This confined and enhanced near‐field leads to an increased absorption cross‐section (Note S2, Supporting Information).^[8c]^ In particular, the field distributions at different cross‐sections (insets in Figure 1g) and near‐field intensities (*I*) confined within the slot region (A_slot_) along the longitudinal direction (Figure 1h) show that the field enhancement was highly focused around the center of the nanoslot, where maximized optical sensitivity is expected. Because the *E*‐field was localized intensively only at the corners of slots, dashed isocurves where most of the total field exists can be illustrated (Figure 1i). The curves were defined at distances of 0.2 (A_0.2_, 50%), 1.0 (A_1.0_, 90%), and 1.3 µm (A_1.3_, 95%) from the edge of corners. This implies that the captured NPs must be positioned precisely within the defined region to be efficiently detected. By doing so, real‐time monitoring of the evolution of the NP assembly using a well‐defined hotspot can also be expected.

### 2.2 Dynamic Monitoring of Nanoparticle Accumulation in Nanoslots by Dielectrophoresis and AC Electro‐osmosis

Under applied AC *E*‐fields, suspended particles in water experience various external forces, which arise from dielectrophoretic (DEP), AC electroosmosis (ACEO) flow, electrothermal flow, gravitation, buoyancy, interparticle Coulomb interaction, and random Brownian motion.^[^ ^14^ ^]^ First, DEP refers to the translational migration of a suspended particle owing to the interaction between the non‐uniform AC field and induced polarization of the particle.^[^ ^15^ ^]^ The time‐averaged DEP force acting on a particle is

**Figure 2:**
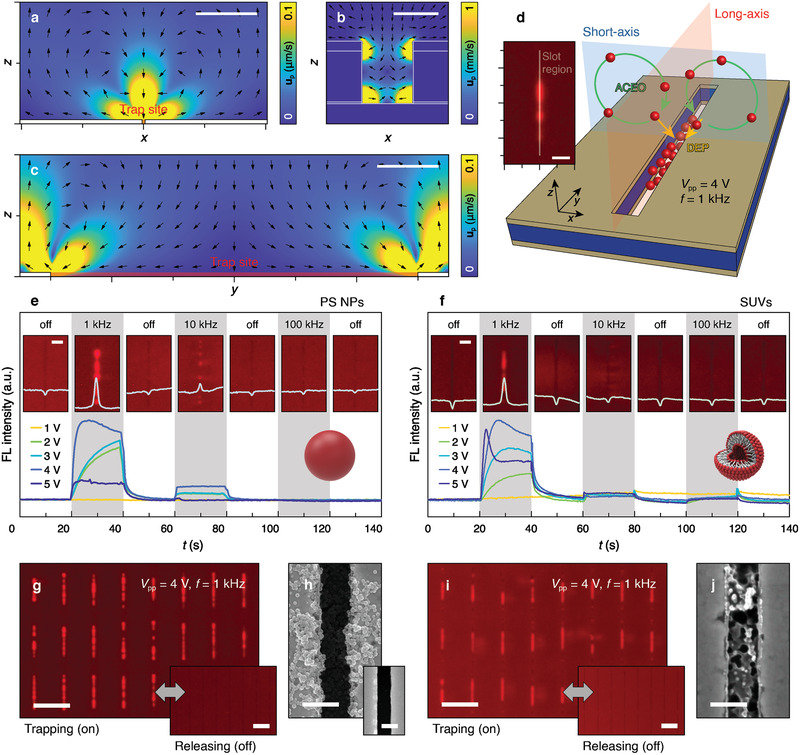
Comprehensive NP dynamics and macroscopic monitoring of NP accumulation. a–c) Calculated axial‐velocity maps showing ACEO driven microvortex generation at each axis (a for *x*‐*z* plane, c for *y*‐*z* plane: Scale bars, 10 µm) together with DEP velocity for NP trapping into nanoslot (b; scale bar, 500 nm) under *V*_pp_ = 4 V and *f* = 1 kHz. d) Conceptual illustration dominant forces in NP localization into nanoslots verified with FL micrograph of trapped PS NPs. Scale bar, 20 µm. e,f) Time‐lapse fluorescence intensity of PS NPs e) and SUVs f) inside nanoslots (*N* = 102) under AC voltage and frequency variations with corresponding FL micrographs of single nanoslot. Scale bars, 10 µm. g–j) FL micrographs of NP trapping/releasing on nanoslot array (Scale bars, 50 µm) and SEM images inside nanoslot h,j, Scale bars, 500 nm) of PS NPs g,h) and SUVs i,j). Inset of SEM for controls without NP accumulation.

(1) FDEP=vp2Re[α]∇Erms2

where *v*_p_, Re[*α*] and **E**_rms_ denote the particle volume, the real part of the particle polarizability (*α*), and the root‐mean‐squared *E*‐field magnitude, respectively. Equation (1) implies that particles are attracted toward (*α* > 0, positive DEP) or repelled from (*α* < 0, negative DEP) the positions of the maximum AC *E*‐field.^[^ ^16^ ^]^ In contrast, ACEO flow is caused by the movements of surface‐attracted ions at the solid–liquid interface under applied AC fields.^[^ ^17^ ^]^ Unlike ACEO, which delivers NPs irrespective of particle characteristics, target‐selective capture/release through DEP is available by controlling the voltage magnitude and frequency.^[^ ^14^ ^]^ Since theoretical simulations and calculations corroborate that DEP and ACEO majorly govern the particle movement under applied AC field, comprehensive dynamics of particles (**u**_p_) described by Langevin equation approximate in terms of **F**_DEP_ and **u**_ACEO_

(2) $$u_{p}\approx u_{\mathrm{ACEO}}+\frac{F_{\mathrm{DEP}}}{6\pi \eta r}$$

in which **u**_ACEO_, *η*, and *r* note for ACEO flow velocity, viscosity, and particle radius, respectively. Considering that other forces are negligibly small (see detail for Note S3, Supporting Information), understanding two major forces (**F**_DEP_ and **F**_ACEO_) and their interplay is key to predicting hydrodynamic NP trajectories over the nanogap array and migrating positions inside the nanoslots. The comprehensive dynamics of NPs were investigated in terms of the particle velocity (**u**_p_), derived from the Langevin equation based on **F**_DEP_ and **F**_ACEO_. The successive delivery of suspended NPs close to nanoslots was possible by the generation of ACEO vortices at the microscale (**Figure** **2a**), then the delivered NPs were snatched into the nanoslot via DEP (Figure 2b). Because the trap site is relatively long—60 µm in the *y*‐direction—while the trap site is approximately 500 nm in the *x*‐direction, there is an axial velocity variation (Figure 2c). The variation in the length disparity, consequently, results in substantial correspondence between the trap site and the sensing hotspot in both the short‐axis (*x*–*z* plane) and long‐axis (*y*–*z* plane) (Figure 2d). The balance between the two dominant forces (**F**_DEP_ and **F**_ACEO_) concentrated and efficiently accumulated NPs into nanoslots in a frequency‐sensitive manner (Note S4, Supporting Information).

To confirm the hypothetical NP dynamics, experimental validations were performed using solutions containing fluorescence (FL)‐labeled 50 nm diameter polystyrene (PS) NPs (10 ppm) and small unilamellar vesicles (SUVs, 1 ppm) (Experimental Section). After placing the particle solutions on the array, AC signals were applied across the electrodes at various frequencies (*f*) and voltage amplitudes (*V*_pp_; peak‐to‐peak voltage) and real‐time FL variations were monitored (Movies M1 and M2, Supporting Information). In this process, time‐dependent FL intensities with respect to different applied voltages and frequencies were measured in 20s intervals of voltage cut off. Resultant FL profiles, acquired from sample number (*N*) of *N* = 102, were analyzed via image‐processing software (Image J) and numerical computing software (Matlab). Methodological details are described in the Experimental Section and Note S5 (Supporting Information). In the monitored micrographs, the stronger FL intensity reflects the larger number of particles. Both PS NPs and SUVs were dominantly populated inside the nanoslots at an AC voltage of *V*_pp_ = 4 V and *f* = 1 kHz, even for ultra‐low particle concentrations (Figure 2e,f). Although the conveying force of **F**_ACEO_ is highly advantageous for long‐range delivery of distant NPs to the vicinity of nanoslot, its balance with **F**_DEP_ is crucial for tweezing the NPs inside nanoslot. According to the simulation of **F**_DEP_ and **F**_ACEO_ under different applied frequencies (Note S4, Supporting Information), the magnitude of **F**_ACEO_ at the slot region was calculated to be weaker than that of **F**_DEP_ under 1 kHz, which allows slow access of traveling NPs around the nanoslots so as to be captured in a facile way. On the contrary for the *f* = 1 kHz, an elevation of applying frequency enhances the magnitude of **F**_ACEO_ while that of **F**_DEP_ is constant. For this reason, snatching of travelling NPs around the nanoslots becomes more challenging as frequency increases (see details for Note S4, Supporting Information). In the same way, concentration of NPs under different applied voltages at *f* = 1 kHz is also examined. The result inform that the dominance of **F**_DEP_ over **F**_ACEO_ for NP accumulation is maintained with escalating *V*_pp_ from 1 V to 4 V, while this become reversed as exceeding 4 V as the **F**_ACEO_ dominantly disrupts NP captures around the nanoslots. Consequently, the AC signal of *V*_pp_ = 4 V and *f* = 1 kHz acts as optimum trapping condition owing to well‐balanced **F**_ACEO_ with **F**_DEP_. This confirms the robustness of our system on contributory roles in NP detection: **F**_ACEO_ for NP delivery, and **F**_DEP_ for NP capture. In addition, material‐dependent NP sorting was observed by frequency control at *f* = 10 kHz. A few PS NPs accumulated inside the nanoslot, while SUVs were weakly held on the periphery of the nanoslot without being trapped inside (insets in Figure 2e,f). FL intensities over numerous nanoslots (*N* = 102, Note 5, Supporting Information), as well as reproducible NP trapping performance under operating voltages, confirmed the device robustness as a large area electric tweezer (Figure 2g–i). Notably, during the accumulation of the SUVs, it can be expected that the agglomerated phospholipids inside the slot region are attributed to electroporation and successive electrofusion between neighboring SUVs above a critical operating voltage.^[^ ^18^ ^]^ In summary, both the simulation and experimental results confirm that a combination of two dominant forces plays a key role in delivering suspended NPs close to nanoslots as well as capturing the delivered NPs into nanoslots.

### 2.3 Stochastic Motion of NPs in Dielectrophoretic Potential Landscape

Microscopic trapping behavior within a single nanoslot was investigated via a rigorous and detailed delineation of NP accumulation. Although it is critical to track the nanophase evolution of NPs to understand its accumulation process at the nanoscale volume, the direct monitoring of NPs at the subwavelength scale is practically impossible. In this case, the electrostatic potential landscape describes the stochastic motions of NPs that move to a position with a lower potential energy, attempting to attain an equilibrium state.^[^ ^19^ ^]^ Assuming that **F**_DEP_ is a conservative force, the work‐energy theorem yields the time‐averaged DEP potential energy of a polarized particle at equilibrium (*U*) as

**Figure 3:**
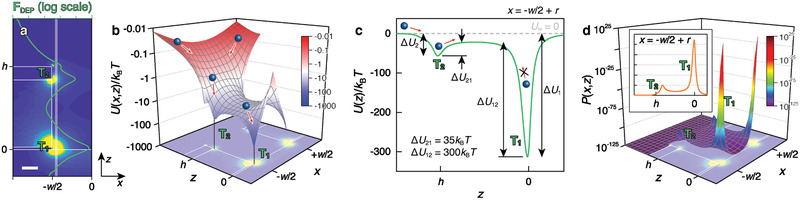
NP dynamics in the potential energy landscape. a) Calculated F_DEP_ distribution (acting on a 50 nm diameter PS NP under *V*_pp_ = 4 V and *f* = 1 kHz) on nanoslot with trapping sites, T_1_ and T_2_. Green curve represents profile along white solid line (*x* = −*w*/2 + *r*). Scale bars = 100 nm. b) Dielectrophoretic potential energy landscape on nanoslot with a schematic of NP migration into T_1_ and T_2_. c) Dielectrophoretic potential energy profile at *x* = −*w*/2 + *r* with schematic of preferred NP migration into T_1_. d) Calculated Boltzmann distribution on nanoslot. (Inset shows profile along *x* = −*w*/2 + *r*).

(3) U=−vp2Re[α]Erms2

with the assumption that *U* is zero at an infinite distance from the nanoslot (*U*_∞_ = 0). The local minima of potential, derived from the calculated side‐view distribution of **F**_DEP,_ were generated at the edges of the electrodes for NP trapping (T_1_ and T_2_, **Figure** **3a**), creating potential energy landscapes with low potential wells at T_1_ and T_2_ (Figure 3b). Considering that the closest NP center of mass was located at distance *r* away from the wall of the nanoslot (*x* = −*w*/2), the potential energy profile at *x* = −*w*/2 + *r* can be regarded as the lowest effective potential. As the depths of the potential wells at T_1_ (Δ*U*_1_ = *U*_∞_ – *U*(T_1_)) and T_2_ (Δ*U*_2_ = *U*_∞_ – *U*(T_2_)) were found to be Δ*U*_1_ = 325 *k*_B_ *T* and Δ*U*_2_ = 60 *k*_B_ *T*, the particles are more likely to be trapped at T_1_ (Figure 3c). Once NPs are captured at T_1_, a large energy barrier from T_1_ to T_2_ (Δ*U*_21_ = 300 *k*_B_ *T*) prevents the captured NPs from escaping, while NPs at T_2_ are likely to traverse into the adjacent T_1_ owing to the relatively shallow barrier in the opposite direction (Δ*U*_12_ = 35 *k*_B_ *T*). For a more intuitive understanding, the probability density functions of the Maxwell–Boltzmann distribution (*P*) can be calculated using

(4) $$P=\frac{1}{N}\exp\left(-\frac{U}{k_{B}T}\right)$$

where *N* denotes the normalization constant and *k*_B_ and *T* denote the Boltzmann constant and temperature, respectively. Regarding the thermodynamic equilibrium of the system, the settlement probability of NPs at T_1_(*P*_1_) was found to be ≈100 orders of magnitude greater than that at T_2_(*P*_2_), where *P*_1_ nearly approaches the delta function (Figure 3d). Thus, we can assume that the captured NPs began to pile up from the bottom edges of the nanoslots (T_1_).

**Figure 4:**
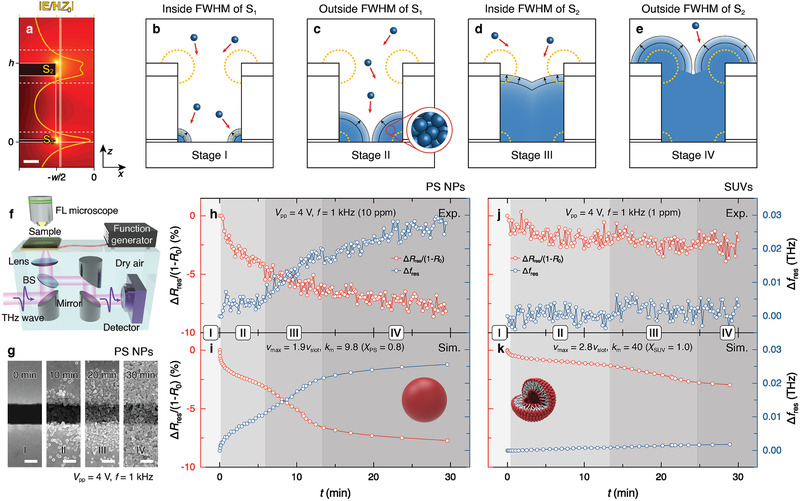
Multistage NP accumulation and real‐time THz optical detection. a) Near‐field distribution of *E*/*HZ*_0_ on nanoslot with optical hotspots, S_1_ and S_2_. Yellow line shows profile along white solid line (*x* = −*w*/2 + *r*). Scale bars = 100 nm. b–e) Schematics of multistage NP accumulation model producing local full wave half minimum (FWHM) of field distribution along the *z*‐axis as trapped NPs piled up. Before‐ b) (Stage I), after crossing the FWHM of S_1_ c) (Stage II), before d (Stage III), and after crossing FWHM of S_2_ e) (Stage IV). f) Experimental setup for THz measurement under simultaneous NP trapping. g) Time‐lapse SEM images of trapped PS NPs on nanoslot every 10 min. h–k) Measured h,j) and calculated i,k) real‐time THz signals (red for Δ*R*_res_/(1 – *R*_0_) and blue for Δ*f*_res_) under accumulation of PS NPs h,i) and SUVs j,k).

The accumulated surface charges at the optical hotspots of the nanoslot antenna (S_1_ and S_2_) interact with the incident electromagnetic wave to provide highly localized field enhancements.^[^ ^10^ ^]^ The distribution of THz field enhancement provides us with the insight that differing field localization results in different sensing signal changes depending on the location of the captured particles (**Figure** **4a**). Taking into account the size of the actual trapped NPs, the effective THz signal changes occur when NPs are localized at the closest center of mass of the NPs (white solid lines in Figure 4a). Because optical sensitivity exponentially decreases with increasing distance from the hotspots, the local full‐wave‐half‐minimums (FWHM; dotted line in Figure 4a) of the field distribution along the *z*‐axis were introduced as criteria to quantify the amount of NPs around hotspots. Considering the times when the NPs pile up and pass through each FWHM line, the accumulation process can be divided into four stages (Figure 4b–e). For example, the NPs initially pile up until they cross the first FWHM line, where the optical signal changes are very sensitive around the first hotspots, S_1_ (Figure 4b; Stage I). As the NPs subsequently accumulate, the optical signal changes less sensitively in the second stage, reflecting the enhanced THz field profile (Figure 4c; Stage II). As more NPs are captured and fill the volume of the nanoslot, crossing another hotspot, S_2_, the optical sensitivity gradually increases again (Figure 4d; Stage III). Finally, after the NPs fill the entire volume of the nanoslot, the additional accumulation of NPs still resulted in small changes in the optical signal and was saturated (Figure 4e; Stage IV). Compared with less sensitive Stages II and IV, accumulation Stages I and III accompany a large spectral shift of resonance owing to amplified signals from the strong interaction between confined field enhancement and target substances.

### 2.4 THz Optical Sensing for Nanoscale Monitoring of Multi‐stage NP Accumulation

The multistage model was experimentally substantiated by monitoring the real‐time reflectance THz spectra changes while trapping 50 nm PS NPs at a low concentration (10 ppm) under an AC voltage of *V*_pp_ = 4 V and *f* = 1 kHz (Figure 4f and the Experimental Section). SEM images (Figure 4g) show the process of NPs piling up inside and around the nanoslots over time and fully covering the outside region in 30 min, as depicted in Figure 4e; Stage IV. Accordingly, the changes of reflectance and frequency as a function of time (*t*) were analyzed in terms of ∆*R*_res_(*t*)/(1 − *R*_0_) = [*R*_res_(*t*) − *R*_0_]/(1 − *R*_0_) and ∆*f*_res_(*t*) = *f*_res_(*t*) − *f*_0_, where *R*_0_ = *R*_res_(*t* = 0) and *f*_0_ = *f*_res_(*t* = 0). As shown in the time‐lapse variations of THz signals, ∆*R*_res_ and ∆*f*_res_ rapidly changed at the initial stage immediately after the voltage was applied (Figure 4h, Stage I), and the values were constantly monitored (Figure 4h, Stage II). Then, the second acceleration of the signal change reappeared (Figure 4h, Stage III) until the growth of the signal change slowed down and saturated with the value, ∆*R*_res_/(1 – *R*_0_) = −7.04% and *∆f*_res_ = 0.024 THz (Figure 4h, Stage IV). The obtained dynamic changes in THz spectra present significant differences with a control experiment without applying a voltage (Supporting Note S6, Supporting Information).

To rationalize the experimental results, numerical simulations were conducted to emulate multiphase NP accumulation and the corresponding optical response within nanoslots (Note S7, Supporting Information). As NPs can be regarded as subwavelength inclusions embedded in the host water medium, the local accumulation of NPs was interpreted as a macroscopically homogeneous medium by adopting the effective medium approach based on the Maxwell–Garnett theory.^[^ ^20^ ^]^ In this process, the partial volumetric parameter of inclusions *χ*_i_ (0 ≤ *χ*_i_ ≤ 1) was introduced to indicate the degree of particle filling, which defines the volumetric ratio of inclusions (*δ*_i_) in the host medium as *δ*_i_ = *χ*_i_·*δ*_max_, where *δ*_max_ is the maximum filling of inclusions $\delta_{\max}=\pi /3\sqrt{2}$. After calculating the far‐field THz reflectance spectra as a function of the geometric parameters, the Michaelis–Menten function (*v*_max_ and *k*_m_ denote the maximum saturation and slope of the curve, respectively) was adopted as a key link between the geometric and time domains, assuming that the amount of NP accumulation increased over time and became saturated (as estimated from Figure 2e,f).^[^ ^21^ ^]^ Interestingly, there are distinct points of inflection at which the trend of the signal changes significantly over time, and the points can be noted as the critical boundaries of each stage in the multistage model, where the THz field profile has local FWHMs in Figure 4a at the same time.

The good correspondence between the measurement and simulation results observed here, relative to that observed for other mono‐stage models (Note S8, Supporting Information), corroborates the multi‐stage accumulation model inside the slot region (Figure 4i). To experimentally confirm the feasibility of our platform for a variety of potential biosensors, a solution of 50 nm SUVs (1 ppm) was tested under the same AC voltage conditions (*V*_pp_ = 4 V and *f* = 1 kHz). The detection of THz signals from underwater SUVs is highly challenging owing to their heterogeneous structures, which comprise a large portion of water inside lipid shells, with a tiny difference in optical properties between water and lipids (Note S9, Supporting Information). Considering the difficulty of selectively sensing the extremely small traces of lipids using any other optical system, the measured THz signal change, 2.35%, in ∆*R*_res_/(1 – *R*_0_) that arose from the ultralow concentration of SUVs is a considerable achievement (Figure 4j). In contrast, the lesser change in ∆*f*_res_ is attributed to the significant similarity on refractive index values between lipids and outside‐/inside‐water of SUVs. It is noted that such highly sensitive and selective detection of the THz signals for target PS NPs and SUVs can be possible only when both the particle trapping sites and sensing sites overlap. In addition, no significant signal change was observed for suspended NPs even at a concentration 10 000 orders of magnitude greater, without the trapping process (Note S6, Supporting Information). With the same simulation procedure adopting the multi‐stage model (Note S10, Supporting Information), it was found that the results match well with those of the experiment, while they exhibit relatively longer stages than those of PS NPs owing to the ultralow concentration of the target analytes (Figure 4k). Consequently, our methodology successfully unraveled the nanoscale dynamics of the particles inside the nanoslot.

## 3 Discussion

Despite substantial efforts to realize surface‐sensitive biosensors via THz spectroscopy, the intrinsic nature of the considerable absorption exhibited by water has been regarded as an immense obstruction. Previous several researches tried to increase the sensing performance, such as the sensitivity, with the assistance of a microfluidic system or an immuno‐sensing surface.^[^8d, 22^]^ Considering the trade‐off between sensitivity increasing using field confinement and absolute signal decreasing by the decreased measurable volume, the need for an actively working sensing platform to offset the water absorption issue still remains. Distinguished from previous underwater approaches, an effective THz signal free from screening by considerable water absorption was realized by the hybridization of electric‐photonic tweezers and nanotechnology. First, nanogap/nanoslot sandwich structures play multi‐functional roles as i) successive NP providers via the generation of convection current through ACEO flows, ii) an NP collector via enhanced **F**_DEP_ generation, and iii) an NP detector for tracing real‐time growth of NPs within a nanovolume. As a result, ACEO‐assisted DEP tweezing allows to significantly concentrate distant NPs at far‐field region as it overcomes limited region and depth of metasurface‐based THz detection. Second, low‐voltage electric tweezing techniques facilitate intact biomolecule trapping under negligible temperature increments, allowing target‐specific sorting without the aid of additional devices or pre/post‐treatment for target selective capture. Third, compared with typical THz transmission measurements, a reflection measurement from the backplane of nanoslots minimizes THz signal loss from bulky water absorption, allowing for highly sensitive optical detection. Finally, beyond the monitoring of 2D NP behavior, which has been accomplished with conventional surface‐enhanced strategies on single‐plane geometries,^[^ ^23^ ^]^ our vertically aligned metasurfaces with bi‐plane electrodes facilitate the tracking of the 3D kinetics of NPs within a nano‐volume.

The dimensional transformation of NP assemblies from the low‐dimensional arrangements of NPs into high‐dimensional assemblies (from 0D dots, 1D lines, 2D sheets, and finally into 3D volumetric aggregates of NPs)^[^ ^24^ ^]^ was successfully monitored by the developed electric‐photonic tweezer. Our results mainly focus on monitoring time‐lapse accumulation progress of particle bodies without considering particle–particle interactions. The measured results can therefore be interpreted as mimicry of various natural nanophase evolution processes, such as NP assembling/clustering^[^1d^]^ and the aggregation/self‐assembly of peptide monomers^[^ ^25^ ^]^ considering physicochemical interactions after biomolecular aggregation. Therefore, this in situ probing technique can be evaluated as a promising tool for resolving the fundamental mechanism of the various physicochemical events of nanomaterials.

To characterize the unique chemical specificity and molecular structures of target analytes, spectrally resolved broadband molecular fingerprints should be analyzed. Although the current research was conducted only at a single resonance condition, the full spectral content of various targets can potentially be retrieved by adopting multi‐resonance approaches that collect optical signals under several on‐demand resonances.^[^ ^26^ ^]^ Additionally, because our platform guarantees a high degree of design flexibility by simply adjusting the device geometry, converging our approach with different types of metasurfaces will facilitate the construction of optical sensors at various frequency regimes. Therefore, our strategies that adopt nanostructures to enhance the sensing capability along with electrically induced dynamic manipulations of the target analytes will create substantial opportunities to develop advanced surface‐sensitive optical biosensors.

## Experimental Section

1

1.1

##### Device Fabrication

The bottom layers of Ti (5 nm)/Au (20 nm) were deposited onto a double‐polished intrinsic silicon (Si) substrate using an electron‐beam evaporator. Then, a 500 nm thick dielectric layer of poly(4‐vinylphenol) (PVP) mixed with a cross‐linking agent, poly(melamine‐*co*‐formaldehyde) methylate (PMF), was spin‐coated and cured. A photoresist (PR) layer was patterned into a reverse pattern of 500 nm wide nanoslots using a maskless lithography system. Next, the Ti (5 nm)/Au (120 nm) layers were deposited, followed by a lift‐off process to arrange the nanoslot patterns on the PVP insulator. Finally, dielectric and bottom metallic layers were sequentially dry‐etched inside the nanoslots to create nanoslot cavities that allowed the external NPs to access the sensing hotspots (see details of the fabrication procedures in Note S1, Supporting Information).

##### SEM Measurement

For the SEM measurement, a field‐emission scanning electron microscope (Nova NanoSEM 200, FEI) equipped with a through‐lens‐detector (TLD) was used. Prior to SEM imaging, the samples were attached to a SEM specimen stub using carbon tape, and a 2 nm thick Pt layer was deposited on the sample surface via ion sputter coating (E‐1045, Hitachi). The measurements were performed under a chamber pressure of 0.1 mbar, accelerating voltage of 10 kV, operating current of 80 pA, and working distance of 5 mm, using the TLD for high‐resolution SEM imaging.

##### Experimental Setup for THz Reflection Measurements

For the measurement of the THz reflection spectra, a standard THz TDS system with a spectral range of 0.2–2.0 THz was employed. A Ti:sapphire laser beam, which generates optical pulses with a central wavelength of 800 nm at a pulse width of 100 fs and repetition rate of 80 MHz, was divided into two components for emission and detection by a beam splitter (BS). The emission beam was irradiated on the surface of a photoconductive antenna to generate a single‐cycle THz electromagnetic pulse. The generated THz pulse wave was normally incident onto the bottom Si interface of the sample through a metal mirror, highly resistive Si BS, and focussed on the metasurface using a polymethylpentene lens. The THz waves reflected from targets were again reflected by the Si‐BS and guided into a ZnTe crystal for electro‐optic sampling detection. Note that the long side of the nanoslot was arranged perpendicular to the polarization of the THz waves. To avoid undesirable absorption optical signals from water vapor, the entire THz TDS system was enclosed in a box with dry air with a humidity of less than 1%. After mounting reference and target samples on the stage of the THz TDS system, *E*_ref_(*t*) and *E*_sam_(*t*), respectively, were obtained. The reflectance of the sample, *R*(*ω*), was obtained using *R*(*ω*) = |*E*_sam_(*ω*)/*E*_ref_(*ω*)|^2^ with the Fourier transform of the measured signal in the time domain. In this research, *E*_ref_(*ω*) was collected from a Au mirror with an identical substrate to the nanoslot sensing device.

##### Preparation of Solutions Containing PS NPs and SUVs

A commercially available solution of suspended 50 nm diameter PS NPs labeled with red fluorescence dye (Ex/Em: 542 nm/612 nm; Fluoro‐Max, Thermo Fisher) was used. The solution of PS NPs was diluted 1000 times with deionized (DI) water to lower the concentration to 10 ppm, resulting in an electric conductivity of 1 µS cm^−1^. Regarding the SUV solution, SUVs containing FL dye‐labeled lipids, Texas Red 1,2‐dihexadecanoyl‐sn‐glycero‐3‐phosphoethanolamine (Ex/Em: 590 nm/610 nm; Thermo Fisher), and 1,2‐dioleoyl‐sn‐glycero‐3‐phosphocholine (Avanti Polar Lipids) were dissolved at a molecular ratio of 1:99 in chloroform at 0.1 mg mL^−1^ for FL visualization. The rapid solvent exchange method^[^ ^27^ ^]^ was employed to change the chloroform to DI water, resulting in spherical lipid balls in the form of multilamellar vesicles. To normalize the size of the lipids into 50 nm diameter unilamellar‐shaped liposomes, the solution was extruded 20 times through polycarbonate membranes with a pore size of 50 nm, resulting in a uniformly sized 50 nm SUV with a final concentration of 1 ppm and an electric conductivity of 1 µS cm^−1^. To measure the electrical conductivity, a conductivity meter (LAQUAtwin EC‐33, Horiba) was used.

##### Experimental Setup for Monitoring AC Field‐Induced Trapping of PS NPs and SUVs

A 100 µm thick polydimethylsiloxane (PDMS) film was prepared by mixing a PDMS elastomer and a curing agent (Sylgard 184, Dow Corning) at a ratio of 10:1 and annealing the mixture at 80 °C overnight. Then, the PDMS film was perforated using a biopsy punch with 4 mm in diameter (MIL3334HH, Miltex) and placed onto the sample to envelop the nanoslot pattern array. A solution volume of 10 µL containing PS NPs/SUVs was dropped on the nanoslot array and covered with a coverslip to prevent water evaporation and to facilitate microscopic observation.^[^ ^28^ ^]^ Electric signals were generated from a waveform generator (SDG2122X, SIGLENT) and systematically controlled using a customized code (Labview 2018, National Instruments). In this process, FL images of particulate materials were obtained using a fluorescence microscope system (LV100ND, Nikon) equipped with a Texas Red FL filter (Ex: 540–580 nm Em: 600–660 nm, Nikon).

##### Statistical Analysis

The time‐lapse FL intensity data from FL microscopic video was quantitated by image‐processing software (Image J) and numerical computing software (Matlab). In this process, FL intensities were acquired from nanoslots of sample sizes *N* = 102. Calculated FL intensity data were represented as mean value in Figure 2e,f (see details for Note S4, Supporting Information).

## Conflict of Interest

The authors declare no conflict of interest.

## Supporting information

Supporting Information

Supplemental Movie 1

Supplemental Movie 2

## Data Availability

The data that support the findings of this study are available from the corresponding author upon reasonable request.
