# Supplementary material for: Nanoscale Terahertz Monitoring on Multiphase Dynamic Assembly of Nanoparticles under Aqueous Environment
Source: Adv Sci (Weinh). 2021 Mar 24;8(11):2004826. doi: 10.1002/advs.202004826 (PMC8188200; doi:10.1002/advs.202004826)
Supplement: Supplementary file 1 — Supporting Information [file ADVS-8-2004826-s001.pdf]

© 2021 Wiley-VCH GmbH

ADVANCED SCIENCE
Open Access

Supporting Information

for *Adv. Sci.,* DOI: 10.1002/advs.202004826

Nanoscale Terahertz Monitoring on Multiphase Dynamic Assembly of Nanoparticles under Aqueous Environment

Eui-Sang Yu, Sang-Hun Lee, Geon Lee, Q-Han Park, Aram J. Chung, Minah Seo, and Yong-Sang Ryu**

Supporting Note 1. Detailed fabrication process

To produce bottom electrode patterns, an image reversal photolithography technique was employed on a double-side-polished intrinsic (resistivity larger than 10,000 $\Omega\cdot cm^{-1}$) 6-inch silicon (Si) wafer that was 500 μm thick (**Figure S1a**). A photoresist (AZ5214E, MicroChemicals) was spin-coated at 3000 rpm for 30 s and annealed at 95 °C for 210 s on a hotplate. Using a mask aligner (MA-6 III, Karl-suss), an image-wise exposure process was conducted using 8 $mW\cdot cm^{-2}$ of ultra-violet (UV; wavelength of 365 nm) light for 6 s through a photomask, followed by a reversal bake for 120 s at 110 °C and 30 s of additional flood exposure, leaving bottom electrode openings after rinsing the wafer in a developer (AZ300 MIF, AZ Electronic Materials) for 40 s (**Figure S1b**). Then, 5/20 nm thick titanium (Ti)/gold (Au) layers were deposited on the patterned wafer using an electron-beam evaporator (EI-5k, ULVAC; **Figure S1c**), and the lift-off process was carried out via 20 min of ultrasonication in acetone and isopropyl alcohol (IPA) (**Figure S1d**). After preparing the patterned bottom electrode layer, the wafer was diced into 2.4 cm × 2.0 cm pieces consisting of four sets of electrodes using a laser ablation Dicer (M-2000, Exitech; **Figure S1e**). A 10 *wt*.% poly(4-vinylphenol) (PVP; 436224, Sigma Aldrich) mixture solution was prepared by dissolving PVP powder in a propylene glycol monomethyl ether acetate solvent (PGMEA; 484431, Sigma Aldrich) with a thermal cross-linking agent, poly(melamine-co-formaldehyde) methylated (PMF; 418560, Sigma Aldrich). A 500 nm thick PVP film was uniformly coated on the entire surface with a sequential spin-coating (3000 rpm for 30 s) and annealing process (100 °C for 10 min), followed by post-annealing at 200 °C for 20 min (**Figure S1f**). To pattern the nanoslot array on the PVP/PMF surface, a maskless photolithography technique was employed. A photoresist (SS03A9, Dongwoo Fine-Chem, 4000 rpm for 40 s) was evenly spin-coated with the aid of a hexamethyldisilazane adhesion promoter (9352-03, J. T. Baker, 2500 rpm for 20 s). After annealing at 95 °C for 1 min 30 s, a computer-aided design (CAD) data-loaded maskless photolithography system (DL-1000 HP, Nano System Solutions) produced 500 nm wide nanoslots under exposure to 80 $mJ\cdot cm^{-2}$ UV light (wavelength of 405 nm). Subsequently, the serial process steps of post-annealing at 110 °C for 90 s, rinsing in a developer (AZ300 MIF, AZ Electronic Materials), and hard-baking at 120 °C for 90 s were conducted to arrange the photoresist layer into inverted nanoslot patterns (**Figure S1g**). To assemble the patterned top layer with a nanoslot array, 5/125 nm thick Ti/Au layers were deposited on the substrate using an electron-beam evaporator (**Figure S1h**) and sonicated in acetone and IPA for 1 h each for lift-off (**Figure S1i**). Prior to etching away the underneath PVP/PMF insulating film and bottom Ti/Au films within the nanoslot region, a patterned photoresist (AZ1512, MicroChemicals) was prepared (**Figure S1j**) for the sequential etching process. O_2 plasma reactive ion etching (RIE) was carried out for removal of the PVP layer under an O_2 gas flow of 100 sccm at an RF power of 150 W and a pressure of 0.1 Torr via a reactive ion etcher (RIE 80 plus, Oxford Instrument) Next, an inductively

coupled plasma (ICP) etcher (PlasmaPro System100 Cobra, Oxford instruments) removed underneath the Ti/Au film until the Si surface was exposed (40 s under Ar and Cl_2 gas flows of 8 and 4 sccm each at an ICP generating power of 1000 W, DC power of 50 W, and pressure of 1.1 Pa, **Figure S1k**). Finally, the fabrication procedures were completed by removing the residual photoresist layer on the sample through ultra-sonication in acetone and IPA for 10 min (**Figure S1l**).

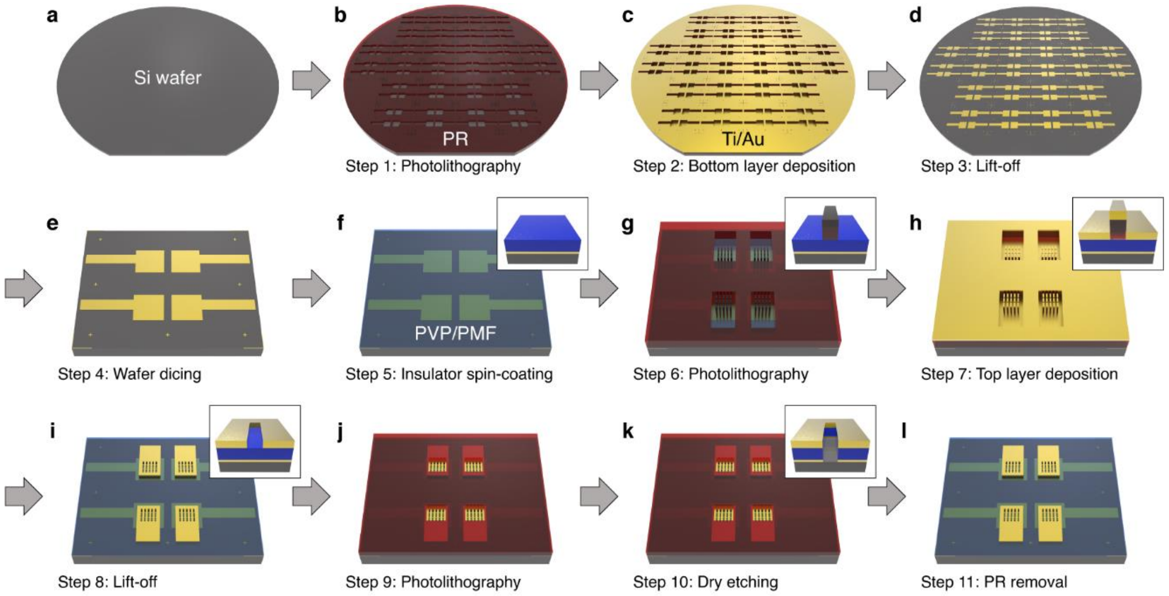

Figure S1. Schematics of the fabrication process. a) Si wafer substrate. **b–d**) Photolithography (**b**), Au E-beam evaporation (**c**), and lift-off (**d**) for bottom electrode pattern. **e**) Four sets of electrodes in single sample after dicing process. **f**) Spin-coating for uniform PVP/PMF film. **g–i**) Maskless photolithography (**g**), Au deposition (**h**), and lift-off (**i**) for 500 nm wide top nanoslot array pattern. **j**) Additional photolithography with exposure of region of interest. **k**) Dry etching for removal of films inside nanoslots. **l**) PR removal leaving four sets of nanoslot arrays in a sample. Insets for schematic illustrations of a nanoslot.

Supporting Note 2. Optical simulation and field enhancement of nanoslots

Numerical model for optical simulation. To investigate the optical characteristics of the nanoslot array, simulations based on a finite element method (FEM) analysis were conducted using COMSOL software (Multiphysics, COMSOL). A three-dimensional (3D) simulation model composed of perforated nanoslot layers between the Si substrate and the surrounding medium was adopted (**Figure S2a**), with its geometric parameters given in **Table S1**. Aside from the Ti adhesion layer, the nanoslot layers consist of bottom Au, insulating PVP/PMF, and top Au layers (**Figure S2b**). To reduce the computational burden, a calculation was conducted only on a single unit cell by imposing periodic boundary conditions on the vertical planar boundaries. By utilizing port conditions and S-parameters for the horizontal boundaries, a plane terahertz (THz) light source, which is perpendicularly polarized to a long-axis of nanoslot within the spectral range of 0.1 to 2.0 THz, was normally incident from the bottom planar boundary.

To implement the dispersive optical response of Au in the frequency range of interest, the Drude model was adopted as follows:

$$\varepsilon(\omega) = \varepsilon_{\infty} - \frac{\omega_{\mathrm{p}}^{2}}{\omega(\omega + i\gamma)}\,. \qquad \text{(S1)}$$

The optical properties of other materials were adopted from the literature and measurements (**Table S2**). While the optical properties of Si, Au, and PVP/PMF were fixed throughout the entire simulation process, those of the surrounding medium and nanoslot region varied depending on the situation. For instance, the optical constants of air, water, and the effective dielectric constants based on the Maxwell-Garnett (MG) approximation (will be described in **Supporting Notes 7** and **10**) were applied to the medium and nanoslot regions to reproduce the atmospheric/aqueous environment and nanoparticle accumulation, respectively.

Field enhancement and enlarged absorption cross-section inside the nanoslot. According to the Fermi golden rule, which describes the transition from one energy eigenstate to another, energy absorption on one molecule is $(2\pi/\hbar)\mu^2E^2\rho(\hbar\omega_0)\hbar\omega_0$ where μ, E, $\rho(\hbar\omega_0)$, and ω_0 are the electric dipole moment of a molecule, electric field of the incident light, density of state at the energy $\hbar\omega_0$, and resonant angular frequency, respectively. Since the molecular absorption cross-section (σ) is derived as $\sigma = (2\pi/\hbar)\mu^2\rho(\hbar\omega_0)\hbar\omega_0(E^2/S)$ based on the Fermi golden rule and becomes larger according to the electromagnetic components of E^2/S, previous research on molecular sensing has defined the absorption enhancement factor (Σ) as[1]

$$\Sigma = E^2/Z_0S = E/Z_0H \qquad \text{(S2)}$$

where S and Z_0 denote the Poynting vector and impedance of free space (377 Ω). To numerically analyze the optical sensing capability of a nanoslot array, the cross-sectional distributions of Σ at resonance conditions were calculated using optical simulations of E and H. While the electric field was enhanced by two orders of magnitude inside the nanoslot (**Figure S3a**), the magnetic field penetrated into the structure without enhancement (**Figure S3b**). As a result, this asymmetric electromagnetic enhancement of E/H facilitates optical sensing inside the nanoslot region.

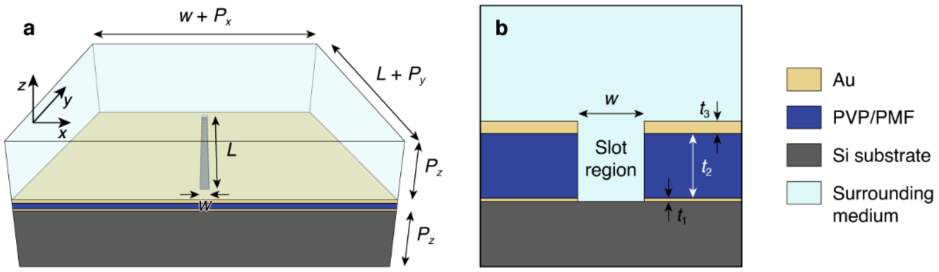

Figure S2. Optical simulation conditions of nanoslot array. a–b) Schematics of numerical model of nanoslot for optical simulation unit cell (**a**) and cross-sectional illustration (**b**).

Parameter	Definition	Values
L	Slot length	60 μm
w	Slot width	500 nm
P_x	x-axial periodic separation	40 μm
P_y	y-axial periodic separation	10 μm
P_z	z-axial separation	5 μm
d_1	Thickness of bottom Au layer	20 nm
d_2	Thickness of insulating PVP/PMF layer	500 nm
d_3	Thickness of top Au layer	100 nm

Table S1. Geometric parameters of optical simulations.

Parameter	Definition	Values
n_{Si}	Refractive index of Si	3.41 [2]
κ_{Si}	Extinction coefficient of Si	0 [2]
$n_{PVP/PMF}$	Refractive index of PVP/PMF	Given in **Figure S17b** (Measured)
$\kappa_{PVP/PMF}$	Extinction coefficient of PVP/PMF	Given in **Figure S17b** (Measured)
n_{air}	Refractive index of air	1
κ_{air}	Extinction coefficient of air	0
n_{water}	Refractive index of water	Given in **Figure S12a** [3]
κ_{water}	Extinction coefficient of water	Given in **Figure S12a** [3]
n_{PS}	Refractive index of polystyrene (PS)	Given in **Figure S13b** [4]
κ_{PS}	Extinction coefficient of PS	Given in **Figure S13b** [4]
n_{DOPC}	Refractive index of 1,2-dioleoyl-sn-glycero-3-phosphocholine (DOPC)	Given in **Figure S17c** (Measured)
κ_{DOPC}	Extinction coefficient of DOPC	Given in **Figure S17c** (Measured)

Table S2. Material properties of optical simulations.

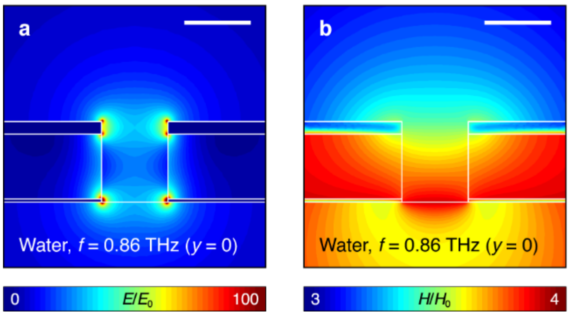

Figure S3. Near-field distribution on nanoslot. a–b) Calculated electric (**a**; E/E_0), and magnetic near-field distribution (**b**; H/H_0) at centre of nanoslot (under water, $f = 0.86$ THz). Scale bars, 500 nm.

Supporting Note 3. Theoretical evaluation of AC field-driven particle motion

Langevin equation for the comprehensive dynamics of particles under a variety of external forces. Under an AC field in a water environment, suspended particles are subjected to various external driving forces. To comprehensively evaluate the dynamics of suspended particles (with radius r and mass m_p) in terms of the velocity of the particle ($\mathbf{u}_p$), the Langevin equation was employed[5]

$$m_p \frac{d\mathbf{u}_p}{dt} = \mathbf{F}_{DEP} + \mathbf{F}_{ACEO} + \mathbf{F}_{ETF} + \mathbf{F}_{grav} + \mathbf{F}_{buoy} + \mathbf{F}_{int} + \xi(t), \quad \text{(S3)}$$

where $\mathbf{F}_{DEP}$, $\mathbf{F}_{ACEO}$, $\mathbf{F}_{ETF}$, $\mathbf{F}_{grav}$, $\mathbf{F}_{buoy}$, $\mathbf{F}_{int}$, and $\xi(t)$ denote the dielectrophoretic force, Stokes drag by AC electro-osmotic flow, Stokes drag by electrothermal flow, gravitational force, buoyant force, interparticle force by Coulomb interaction, and Brownian random force, respectively. According to the simulations and calculations on 50 nm diameter PS nanoparticles (NPs) presented below, $\mathbf{F}_{DEP}$ and $\mathbf{F}_{ACEO}$ are dominant, while the others are negligible ($\mathbf{F}_{int}$ is negligible under a low concentration of particles, such as in our experimental cases of 1 and 10 ppm). Considering that the relaxation time of the particle ($\tau = m/6\pi\eta r \approx 1.6 \times 10^{-10}$ s, where η is the dynamic viscosity of the medium) was found to be much shorter than the typical time of experimental observation (t), the transient regime at a short time scale ($t << \tau$) quickly saturates into a viscous regime at a time scale longer than τ ($t >> \tau$). Therefore, **Equation S3** saturates into the terminal $\mathbf{u}_p$ in the viscous regime and becomes simplified as[6]

$$\mathbf{u}_p = \left(\mathbf{u}_{ACEO} + \frac{\mathbf{F}_{DEP}}{6\pi\eta r}\right)\left(1 - e^{-t/\tau}\right) \approx \mathbf{u}_{ACEO} + \frac{\mathbf{F}_{DEP}}{6\pi\eta r}, \quad \text{(S4)}$$

with the assumption that $\mathbf{u}_p$ is initially 0. Hence, the motions of particles under various AC signals are simply described in terms of combination and their interactive balance between $\mathbf{F}_{DEP}$ and $\mathbf{F}_{ACEO}$.

To estimate the effect of various external forces, the electrokinetic and electrohydrodynamic properties of the materials in **Table S3** were utilized throughout the entire process. In particular, for the characterization of forces including $\mathbf{F}_{DEP}$, $\mathbf{F}_{ACEO}$, and $\mathbf{F}_{ETF}$, simulations based on FEM were carried out using COMSOL Multiphysics. In this simulation process, a two-dimensional (2D) numerical model emulating a single nanoslot unit was adopted with the geometric parameters described in **Table S4**.

Simulation of dielectrophoresis (DEP). The time-averaged DEP force ($\mathbf{F}_{DEP}$) acting on a spherical particle (radius of r) suspended in a medium under an AC E-field (applied peak-to-peak voltage amplitude of V_{pp} and frequency of f) is defined as

$$\mathbf{F}_{DEP} = \frac{v_p}{2}\mathrm{Re}\ \alpha\ \nabla\mathbf{E}_{rms}^2 , \tag{S5}$$

where v_p, Re[α], and $\mathbf{E}_{rms}$ denote the volume of the particle ($v_p = 4\pi r^3/3$), the real value of particle polarizability (α), and the root-mean-squared E-field magnitudes. In particular, polarizability is given by

$$\alpha = 3\varepsilon_0\varepsilon_m\left(\frac{\varepsilon_p^* - \varepsilon_m^*}{\varepsilon_p^* + 2\varepsilon_m^*}\right), \tag{S6}$$

where ε_0, ε^*_p, and ε^*_m are the permittivity of free space, complex permittivities of the medium ($\varepsilon^*_m = \varepsilon_m - i(\sigma_m/\omega)$) and particles ($\varepsilon^*_p = \varepsilon_p - i(\sigma_p/\omega)$), respectively. Note that ε_m, σ_m, ε_p, σ_p, i, and ω indicate the permittivities and electrical conductivities of the medium and particle, imaginary unit ($\sqrt{-1}$), and angular frequency ($\omega = 2\pi f$), respectively. The term inside the brackets of **Equation S6** is known as the Clausisus-Mossotti (CM) factor ($f_{CM}(\omega)$)[5], which represents the effective polarization of suspended particles under an E-field. See **Figure S4a** for a schematic illustration of the simulation model for DEP. Note that root-mean-squared voltage amplitudes (V_{rms}) were utilized for a time-averaged solution.

Simulation of AC electro-osmosis (ACEO). On the surface of electrodes in an ionic environment, an attraction of counterions against the applied voltage forms a thin layer of an electrical double layer (EDL) at the interface between the bulky aqueous medium and the solid surface. Whereas immobile counterions are attracted within the Stern layer (the thickness of one hydrated ionic diameter, 0.3 nm) where crowded immobile counterions are located in front of the solid surface, the diffuse layer consists of alternating layers of mobile counterions and coions. In the presence of the AC field, the ions at the diffuse layer move along the solid surfaces owing to the tangential components of the electric fields, thus generating flow at the boundary of the electrodes, called the long-range order of ACEO flow, which can move floating analytes and nanoparticles along the streamline of the flow.

For the analytic validation of electrohydrodynamic behavior based on EDL, the Stern model[7] was adopted. Assuming a quasi-equilibrium thin double layer approximation that the voltage drop is linear across the diffuse layer, the boundary condition of potential (ϕ) right over the EDL on the surface of the electrode (parallel in the x-axial direction) is given by[8]

$$\sigma\frac{\partial\phi}{\partial y} = i\omega C_{DL}(\phi - V_0) , \tag{S7}$$

where C_{DL} and V_0 refer to the capacitance per unit area of the EDL and the potential applied to the electrode, respectively. The tangential component of the time-averaged fluid speed ($\langle u \rangle$) at the EDL/medium interface, which arises from the interaction of the EDL and E fields, is represented as[9]

$$\langle u \rangle = -\frac{\varepsilon}{4\eta}\Lambda\frac{\partial}{\partial x}\left|\phi - V_0\right|^2 \tag{S8}$$

based on the Helmholtz-Smoluchowski equation[10]. In **Equation S8**, Λ denotes the capacitive ratio between the total EDL and the diffuse layer, $\Lambda = C_{DL}/C_d = C_s/(C_s + C_d)$, in which C_s and C_d are capacitances of Stern and diffuse layers, and assumed to be 0.2 considering a low conductive environment[8]. Then, the bulk fluidic flow of $\mathbf{u} = (u, v)$, in which u and v denote the horizontal and vertical velocities, respectively, arising from the slip velocity at the solid interface over the electrodes, was calculated using the Navier–Stokes equation,

$$\eta\nabla^2\mathbf{u} - \nabla p = 0, \tag{S9}$$

and mass conservation ($\nabla\cdot\mathbf{u} = 0$), where p is the pressure. See **Figure S4b** for a schematic illustration of the simulation model for ACEO. For the calculation of the Stokes drag force induced by ACEO flow ($\mathbf{F}_{ACEO}$), the definition of the Stokes drag force, $\mathbf{F} = -6\pi\eta R(\mathbf{u}_p - \mathbf{u}_m)$, where $\mathbf{u}_m$ represents fluid velocity[6], was used by replacing $\mathbf{u}_{ACEO}$ with $\mathbf{u}_m$ with an initial condition of $\mathbf{u}_p = 0$, indicating that electric potential has just been applied and the particle is at rest[11].

Simulation of electrothermal flow (ETF). ETF is a fluidic motion that arises from the non-uniform thermal distribution induced by the applied AC fields. Based on the simulation results of **E**, the Joule heating effect was investigated in terms of absolute temperature (T) using the simplified energy balance equation[12],

$$k\nabla^2 T + \sigma\mathbf{E}^2 = 0. \tag{S10}$$

Because the temperature gradient (∇T) from localized heating results in the gradients of permittivity ($\nabla\varepsilon$) and conductivity ($\nabla\sigma$) of the medium[12]; $\nabla\varepsilon = (\partial\varepsilon/\partial T)\cdot\nabla T$ and $\nabla\sigma = (\partial\sigma/\partial T)\cdot\nabla T$, the combined electric force ($\mathbf{F}_e$) of Coulomb and dielectric forces arise[13]:

$$\mathbf{F}_e = \frac{1}{2}\cdot\frac{\varepsilon(\alpha-\beta)}{1+(\omega\varepsilon/\sigma)^2}\;\nabla T\cdot\mathbf{E}\;\mathbf{E} - \frac{1}{4}\varepsilon\alpha\left|\mathbf{E}\right|^2\nabla T\,, \tag{S11}$$

where $\alpha = (\partial\varepsilon/\partial T)/\varepsilon$ = -0.4 %·K^{-1} and $\beta = (\partial\sigma/\partial T)/\sigma$ = 2.0 %·K^{-1}, respectively[14]. Neglecting the inertial term, the flow velocity ($\mathbf{u}$) was calculated using the incompressible Navier–Stokes equation with external forces ($\mathbf{F}_e$)[15],

$$\eta\nabla^2\mathbf{u} - \nabla p + \mathbf{F}_e = 0\,, \tag{S12}$$

with the equation of mass conservation, $\nabla \cdot \mathbf{u} = 0$. See **Figure S4c** for a schematic illustration of the simulation model for Joule heating and ETF. Note that $\mathbf{F}_{ETF}$ was calculated using the definition of Stokes drag presented above. The simulation results (**Figure S5**) indicate that the temperature increases to less than 0.001 K, so $\mathbf{F}_{ETF}$ is negligible (in the order of 10^{-19} – 10^{-23} N) when compared with $\mathbf{F}_{DEP}$ and $\mathbf{F}_{ACEO}$ (in the order of 10^{-16} – 10^{-13} N, **Supporting Note 4**).

Calculation of gravitation and buoyancy. $\mathbf{F}_{grav}$ and $\mathbf{F}_{buoy}$ acting on a particle are given by

$$\mathbf{F}_{grav} + \mathbf{F}_{buoy} = v\Delta\rho\mathbf{g}, \tag{S13}$$

where $\mathbf{g}$ and $\Delta\rho$ denote the gravitational acceleration (9.81 $m \cdot s^{-2}$) and density difference between the particle and medium, respectively; $\Delta\rho = \rho_p - \rho_m$ (ρ_p and ρ_m for the density of the particle and medium, respectively). Under a water environment, the forces exerted on the 50 nm diameter PS particles were calculated to be 3.4×10^{-20} N, which are negligible compared with $\mathbf{F}_{DEP}$ and $\mathbf{F}_{ACEO}$ (in the order of 10^{-16} – 10^{-13} N, **Supporting Note 4**).

Effect of Brownian motion and diffusion. To estimate the effect of Brownian motion and diffusion on suspended particles under the simultaneous interaction of advective and diffusive transportation, the Péclet number for mass transfer (Pe) was introduced. Because the term is defined as the ratio of the rate of the advection of a physical quantity by the flow to the rate of diffusion of the identical quantity assisted by an appropriate gradient, the motions of suspended particles are primarily determined by external driving forces for Pe >> 1, while Brownian diffusion is dominant for Pe << 1. On the basis of the mass diffusion coefficient (D) derived from the Stokes–Einstein relationship ($D = k_B T/6\pi\eta r$, where k_B denotes the Boltzmann coefficient), Pe for mass transfer is defined as

$$\mathrm{Pe} = Lu/D, \tag{S14}$$

where L and u are the characteristic length of the system and the speed of the fluid, respectively. Because the simulation results showed that magnitudes of $\mathbf{u}_p$ were approximately 10^{-4} $m \cdot s^{-1}$ (**Supporting Note 4** and **Main Figure 2**), the Pe of the system ($L \sim 40$ μm) with 50 nm diameter PS particles was calculated to be Pe >> 1 (Pe ~ 10^4), indicating that stochastic term $\xi(t)$ is negligible[16].

Parameter	Definition	Values
ε_0	Permittivity of free space	8.85×10^{-12} F·m^{-1}
ε_{water}	Permittivity of water	$80 \cdot \varepsilon_0$ [17]
σ_{water}	Electrical conductivity of water	10^{-4} S·m^{-1} (Measured)
k_{water}	Thermal conductivity of water	0.6 W·K^{-1}·m^{-1} [18]
ε_{Au}	Permittivity of Au	-
σ_{Au}	Electrical conductivity of Au	4.9×10^{7} S·m^{-1} [19]
k_{Au}	Thermal conductivity of Au	318 W·K^{-1}·m^{-1} [19]
$\varepsilon_{PVP/PMF}$	Permittivity of PVP/PMF	$4.7 \cdot \varepsilon_0$ [20]
$\sigma_{PVP/PMF}$	Electrical conductivity of PVP/PMF	10^{-13} S·m^{-1} [21]
$k_{PVP/PMF}$	Thermal conductivity of PVP/PMF	0.2 W·K^{-1}·m^{-1} [22]
ε_{Si}	Permittivity of Si	11.7 [23]
σ_{Si}	Electrical conductivity of Si	1.28×10^{-3} S·m^{-1} (Calculated from [24] and [25])
k_{Si}	Thermal conductivity of Si	1.3 W·K^{-1}·m^{-1} [26]
ε_{glass}	Permittivity of glass (cover slip)	$4.6 \cdot \varepsilon_0$ [27]
σ_{glass}	Electrical conductivity of glass (cover slip)	10^{-11} S·m^{-1} [28]
k_{glass}	Thermal conductivity of glass (cover slip)	1 W·K^{-1}·m^{-1} [19]
η_{water}	Dynamic viscosity of water	8.9×10^{-4} Pa·s [29]
ρ_{water}	Density of water	0.997 g·ml^{-1}
ρ_{PS}	Density of PS	1.05 g·ml^{-1}

Table S3. Material properties of electrokinetic and electrohydrodynamic simulations

Parameter	Definition	Values
L	Slot length	60 μm
w	Slot width	500 nm
P_x	x-axial periodic separation	40 μm
P_y	y-axial periodic separation	10 μm
d_1	Thickness of bottom Au layer	20 nm
d_2	Thickness of insulating PVP/PMF layer	500 nm
d_3	Thickness of top Au layer	100 nm
d_{sub}	Thickness of substrate	500 μm
d_{water}	Thickness of water layer	100 μm
d_{cover}	Thickness of cover slip	100 μm

Table S4. Geometric parameters of electrokinetic and electrohydrodynamic simulations

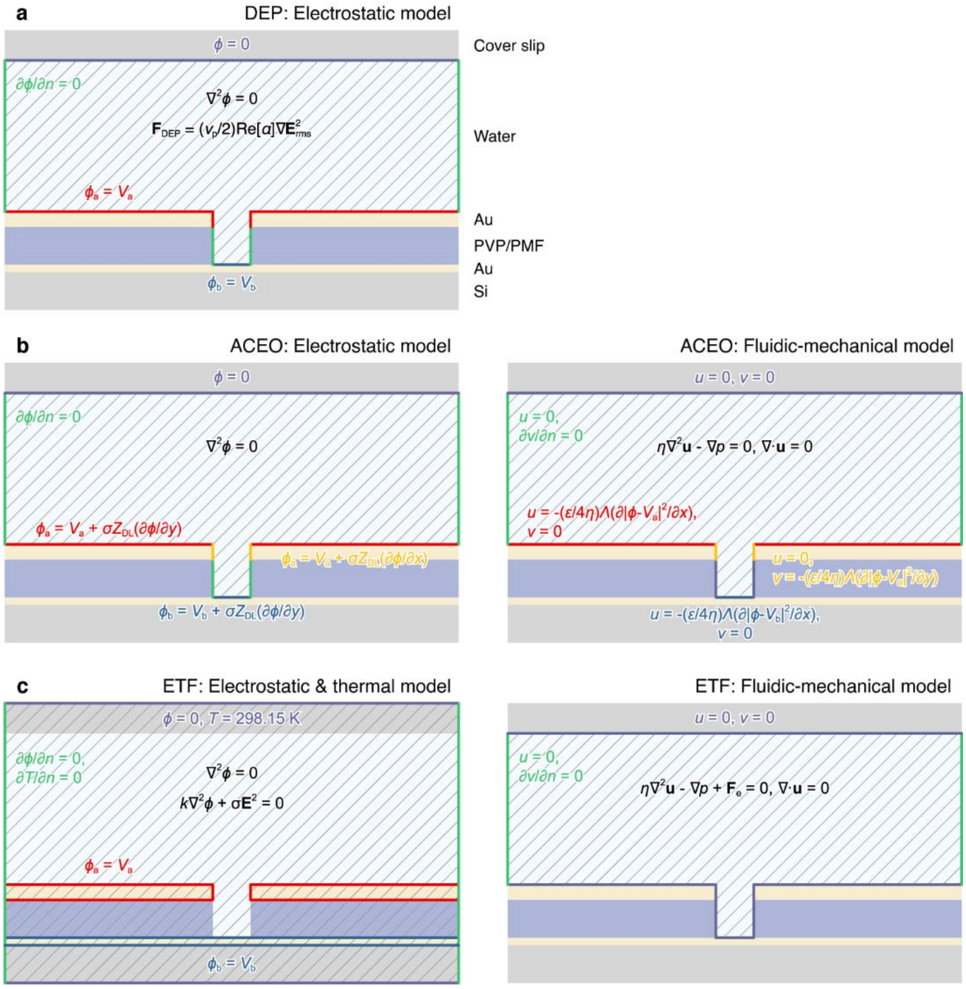

Figure S4. Simulation models for DEP, ACEO, and ETF. a–**c**) Schematic illustrations of boundary and bulk conditions on simulation models of DEP (**a**), ACEO (**b**; left for electrostatic model and right for fluidic-mechanical model), and ETF (**c**; left for electrostatic/thermal model and right for fluidic-mechanical model).

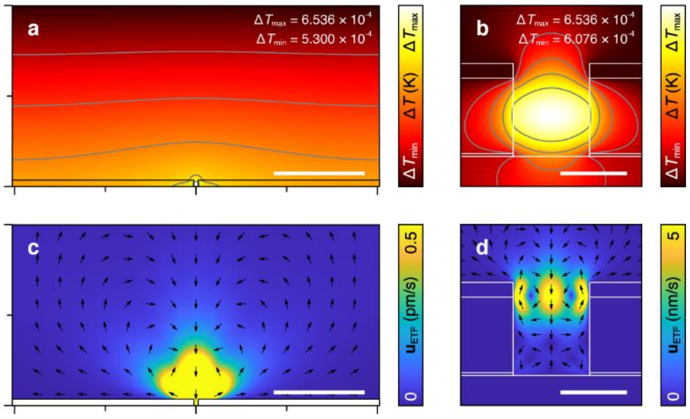

Figure S5. Simulation of Joule heating and ETF on nanoslot array. a–**d**) Calculated distribution of ΔT (**a**, **b**) and $\mathbf{u}_{ETF}$ (**c**, **d**) outside (**a**, **c**; Scale bars, 10 µm) and inside nanoslot (**b**, **d**; scale bars, 500 nm) under V_{pp} = 4 V and f = 1 kHz. Note that arrows which indicate directions of $\mathbf{u}_{ETF}$ are normalized.

Supporting Note 4. Simulation of ACEO and DEP under various AC voltage conditions.

Considering that other forces are negligible in particle movements in liquids (**Supporting Note 3**), we now focus on two dominant forces, $\mathbf{F}_{DEP}$ and $\mathbf{F}_{ACEO}$, to predict the trajectories and positions of NPs under AC fields. As described in the Langevin equation with velocity of the particle ($\mathbf{u}_p$), the spatiotemporal movements of NPs over the nanoslots can be described in terms of velocity; thus, we calculated both the flow distributions of the $\mathbf{u}_{ACEO}$ and $\mathbf{u}_{DEP}$ (**Figures S6a**–**S6c**) to predict the trajectories and positions of NPs. For f = 1 kHz, NPs are conveyed by long-range circulating ACEO micro-vortices outside the nanoslot region where ACEO is dominant over the DEP (first row in **Figure S6a**). However, as the traveling NPs approach the nanoslots, the NPs are forced toward the edges of the nanoslots where the maximum E-field gradient occurs (first row in **Figure S6b**) by the superior DEP forces ($\mathbf{F}_{DEP} > \mathbf{F}_{ACEO}$). As a result, a large amount of NP accumulation is expected inside the nanoslot at f = 1 kHz (first row in **Figure S6c**). However, with increasing applying f, the dominant forces become reversed; thus, a faster ACEO velocity now disrupts particle trapping in the nanoslot (middle rows for f = 10 kHz, and bottom rows for 100 kHz in **Figures S6a**–**S6c**). Because the results clearly demonstrate the role of the balance between DEP and ACEO in NP dynamics, an AC signal of 1 kHz can be regarded as an optimum trapping condition, and the experimental results in the main manuscript (**Main Figures 2e** and **2f**) sufficiently rationalize the simulation results. In summary, under optimum NP capture conditions, ACEO contributes to the successive delivery of remote NPs toward the vicinity of the nanoslot region, while DEP captures the adjacent NPs inside the nanoslot when they pass by. The magnitudes of $\mathbf{u}_{DEP}/\mathbf{u}_{ACEO}$ and $\mathbf{F}_{DEP}/\mathbf{F}_{ACEO}$ were calculated to be in the order of 10^{-7} – 10^{-4} m/s and 10^{-16} – 10^{-13} N, respectively. Note that the conversion of the physical parameters between $\mathbf{u}_{DEP}/\mathbf{u}_{ACEO}$ and $\mathbf{F}_{DEP}/\mathbf{F}_{ACEO}$ was conducted based on the definition of the Stokes drag force described above (**Supporting Note 3**).

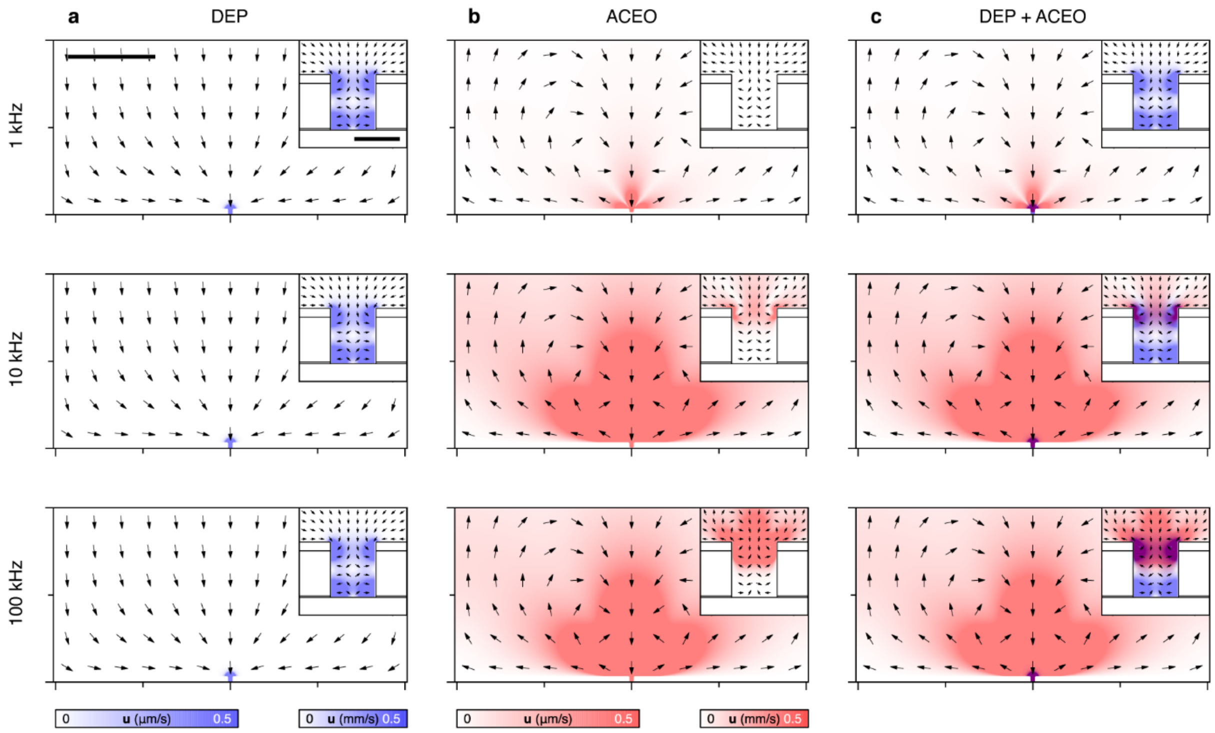

Figure S6. Simulation of DEP and ACEO on nanoslot array. a–**c**) Calculated distributions of $\mathbf{u}_{\mathrm{DEP}}$ (**a**), $\mathbf{u}_{\mathrm{ACEO}}$ (**b**), and their overlapped images (**c**) outside and inside nanoslot (insets; scale bars, 500 nm) under V_{pp} = 4 V and f = 1 kHz. Scale bars, 10 μm. Note that arrows that indicate directions of $\mathbf{u}_{\mathrm{DEP}}$ and $\mathbf{u}_{\mathrm{ACEO}}$ are normalized.

Supporting Note 5. Image processing and analysis of fluorescent (FL) microscopic images.

Time-lapse analysis of FL intensities. After obtaining FL microscopic videos of NP captures during voltage application, particle trapping toward nanoslots by AC fields was systematically analyzed using image-processing software (ImageJ, National Institutes of Health, USA) and numerical computing software (Matlab, Mathworks). Because the population of captured NPs is proportional to the FL intensities of the nanoslots, time-dependent 2D FL intensity was achieved from the image frames of video and defined as $\mathbf{I}(x,y,t)$. Note that t, x, and y denote time and 2D axial components, respectively, while each component in the matrix represents the FL intensity value of the pixelated image. To monitor the FL variation of the nanoslots, $\mathbf{I}(x,y,t)$ were simplified by averaging the y-axial FL intensities (yellow box in **Figure S7a**) and plotted averaged FL profiles $I_p(x, t)$ with time variations. In this plot profile, we set the nanoslot regions where the local FL minimum intensity was monitored and defined as the positions of the nanoslots, x_m^{slot}, where m denotes the term number of nanoslots (**Figure S7b**). Then, time-lapse FL intensities on local minima were averaged into a single plot, which is given as

$$I_0(t) = \sum_m I_p(x = x_m^{slot}, t) / N \,, \quad \text{(S15)}$$

where N is the total number of analyzed nanoslots (N = 102). As shown in the time-lapse plot of **Equation S15** (blue line in **Figure S7c**), the graph shows a gradual decrease in the overall FL intensity owing to FL photobleaching under white light illumination. With the simple assumption that FL intensity decreases linearly over time, linear approximation into $L(t)$ was adopted to model the FL photobleaching (red dotted line in **Figure S7c**). Finally, the photobleaching effect was excluded by subtracting $L(t)$ from **Equation S15** as follows:

$$I(t) = \sum_m I_p(x = x_m^{slot}, t) / N - L(t) \quad \text{(S16)}$$

in order to monitor FL variation above nanoslots (**Figure S6d**).

Large-area analysis of FL intensities. To evaluate large-area uniformity and confirm the device robustness of the optical sensors, guaranteeing consistent signal reproducibility, FL microscopic videos of NP captures under voltage application were numerically analyzed over a sufficiently large total number of N = 102. By image processing the FL micrographs of the nanoslot array, FL intensities of the m-th nanoslot were defined as $\mathbf{I}_m(x,y,t)$ (**Figure S8a**). The N number of $\mathbf{I}_m(x,y,t)$ was averaged into a single time-dependent matrix calculated as follows:

$$\mathbf{I}_{avg}(x,y,t) = \sum_m^N \mathbf{I}_m(x,y,t) / N \,. \quad \text{(S17)}$$

The calculated results were also presented as an image sequence of a single nanoslot (**Figure S8b**). To evaluate the uniformity of trapping performance on array, time-lapse matrices of standard deviations, $\sigma_m(t)$, were calculated as follows:

$$\sigma_m(t) = \sqrt{\frac{1}{XY}\sum_x^X\sum_y^Y\left[\frac{\mathbf{I}_m(x,y,t)-\mathbf{I}_{\text{avg}}(x,y,t)}{\mathbf{I}_{\text{avg}}(x,y,t)}\right]^2}\,, \tag{S18}$$

where x, y, X, and Y are integers that represent x- and y-axial pixel positions, respectively. Subsequently, $\sigma_m(t)$ is represented as histograms with a Gaussian normal distribution fit, which is given by

$$g(x) = \frac{1}{\sigma_\text{n}\sqrt{2\pi}}\exp\left[-\frac{(x-\mu)^2}{2\sigma_\text{n}^2}\right], \tag{S19}$$

where μ and σ_n denote the mean value and standard deviation of the Gaussian function (**Figure S8c**). The time-lapse variation of σ_n was found to be about 1.2×10^{-3} and within the range of 0.8×10^{-3} – 2.2×10^{-3} (**Figure S8d**). This result implies that 99 % of the nanoslots exhibit FL intensity variations within ± 1 %, demonstrating the large-area uniformity and device robustness of our platform as a promising optical sensor.

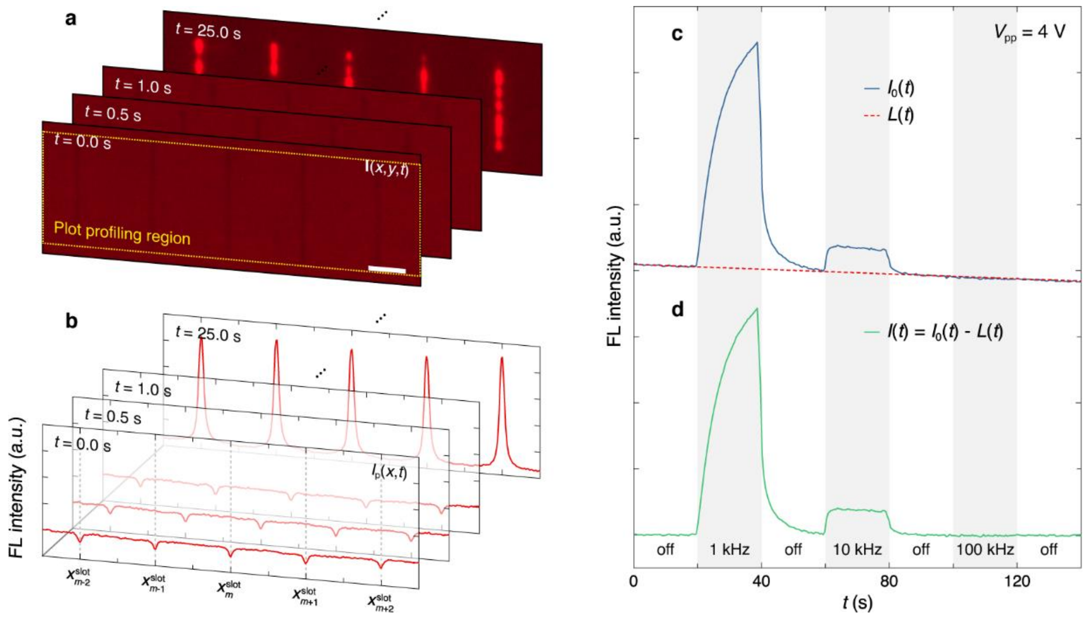

Figure S7. Time-lapse analysis of FL intensity on the nanoslot array. a–b) Time-lapse FL micrographs (**a**) and corresponding FL intensity profiles (**b**) of nanoslot array. Scale bar, 20 μm. **c–d**) Averaged FL profiles as a function of time (**c**; blue curve) measured at local minima (x_n^{slot}) including photobleaching effect (**c**; red dotted line), and after removal of photobleaching effect (**d**; green curve) under AC field variation. In this process of analysis, a solution of 50 nm diameter PS NP was used with AC voltages of V_{pp} = 4 V and f = 1, 10, and 100 kHz.

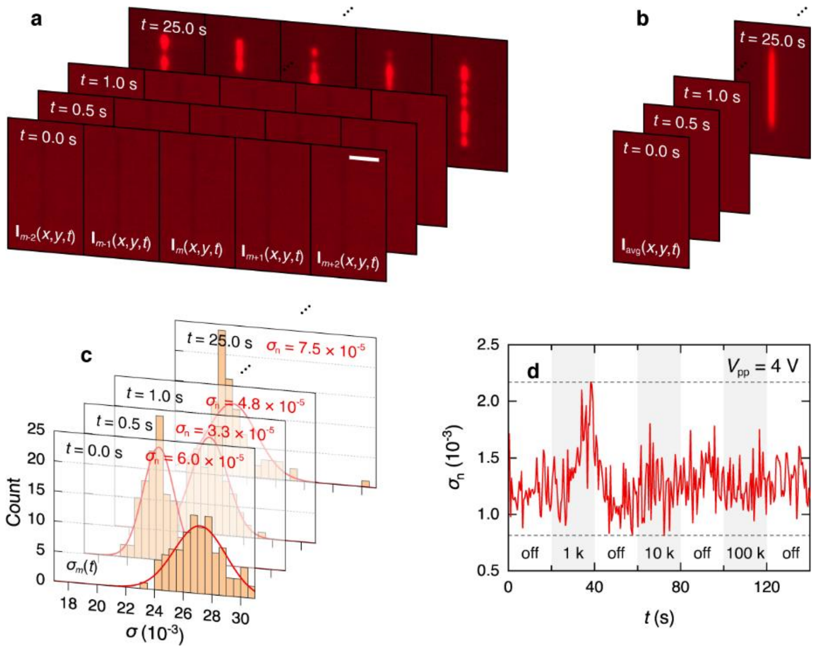

Figure S8. Large-area analysis of FL intensities on nanoslot array. a–**b**) Time-lapse FL micrographs (**a**) and averaged FL distribution (**b**) of nanoslot array. Scale bar, 20 µm. **c**–**d**) Time-lapse histograms of $\sigma_m(t)$ with Gaussian normal distribution fit (**c**) and variation of $\sigma_\mathrm{n}(t)$ (**d**) under AC field variation. In this process of analysis, a solution of 50 nm diameter PS NP was used with AC voltages of V_pp = 4 V and f = 1, 10, and 100 kHz.

Supporting Note 6. Time-lapse measurements of reflectance spectra and control experiments.

Time-lapse measurements of reflectance spectra during NP accumulation. After mounting the devices on a sample stage of reflective THz time-domain spectroscopy (TDS), solution droplets containing PS NPs and SUVs were placed on the patterned nanoslot array. Then, immediately after applying the AC electric signals of V_{pp} = 4 V and f = 1 kHz, reflectance spectra were sequentially measured for 30 min with a time step of 12 s (**Figures S9a** and **S9b**). Because the measured THz resonance spectra vary as NP accumulation proceeds, time-lapse variations of THz reflectance spectra were analyzed in terms of changes in the THz reflectance ($\Delta R_{res}/(1 - R_0)$) and frequency shift (Δf_{res}), calculated as follows:

$$\Delta R_{res}/(1 - R_0) = [R_{res}(t) - R_0]/(1 - R_0), \text{ and } \Delta f_{res} = f_{res}(t) - f_0, \quad \text{(S20)}$$

where R_0 and f_0 denote the initial resonance condition of $R_{res}(t = 0)$ and $f_{res}(t = 0)$ at the time of voltage application (**Main Figure 4**).

Control Experiment I: Time-lapse THz signal from pure deionized (DI) water. To rationalize the THz signal from the accumulation process of PS NPs and SUVs, control experiments using pure DI water were carried out under the same conditions (**Figure S10a**). Compared with those of PS NPs and SUVs (**Main Figure 4**), $\Delta R_{res}/(1 - R_0)$, and Δf_{res} of water were also found to be constant over time with signal fluctuations of ±1.41 % and ±0.0035 THz, respectively (**Figure S10b**).

Control experiment II: THz signal from particle solutions without accumulation. As another control experiment, THz signals from solutions of different concentrations of NPs were collected without an accumulation process (**Figure S11a**). Solutions of PS NPs and SUVs were prepared for different concentrations— 1, 10, 100, 1000, and 10000 ppm—and THz measurements were performed without applying voltage. Irrespective of solution concentrations, $\Delta R_{res}/(1 - R_0)$ and Δf_{res} from the solutions were also found to be constant within the range of signal fluctuations, irrespective of solution concentrations (**Figure S11b**). Even with a very high concentration of NPs, no meaningful signal change was obtained. The results imply that controlling the accumulation of NPs plays a key role in THz optical sensing.

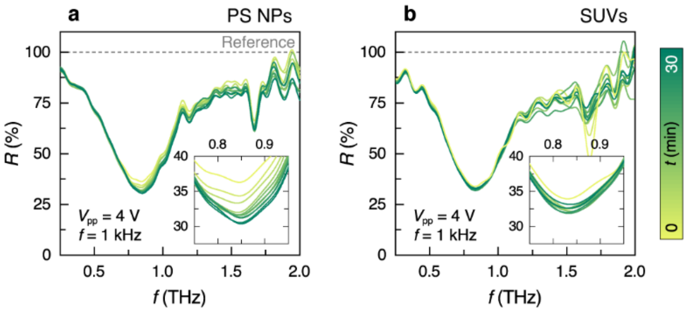

Figure S9. THz reflectance spectra of nanoslot arrays under particle accumulation. a–c) Experimental time-lapse THz reflectance spectra on nanoslot array, measured using solutions of PS NPs (**a**) and SUVs (**b**) under AC voltage of V_{pp} = 4 V and f = 1 kHz.

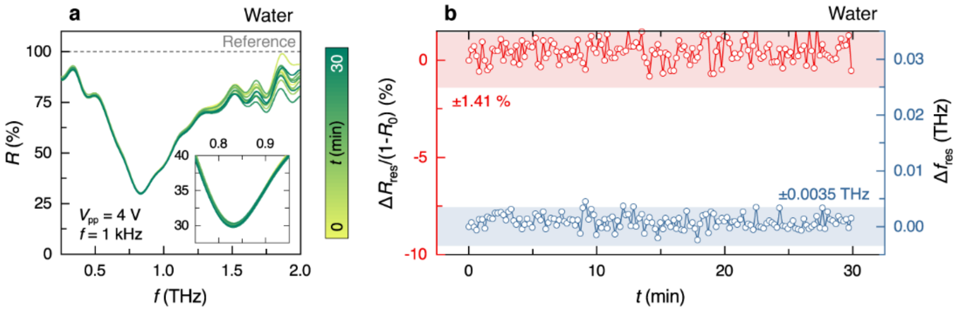

Figure S10. Control experiment I: THz signal detection under DI water. a–b) Experimental time-lapse THz reflectance spectra (**a**) and time-lapse variation of $\Delta R_{res}/(1\text{-}R_0)$ and Δf_{res} (**b**) of nanoslot array measured using pure DI water under AC voltage of V_{pp} = 4 V and f = 1 kHz.

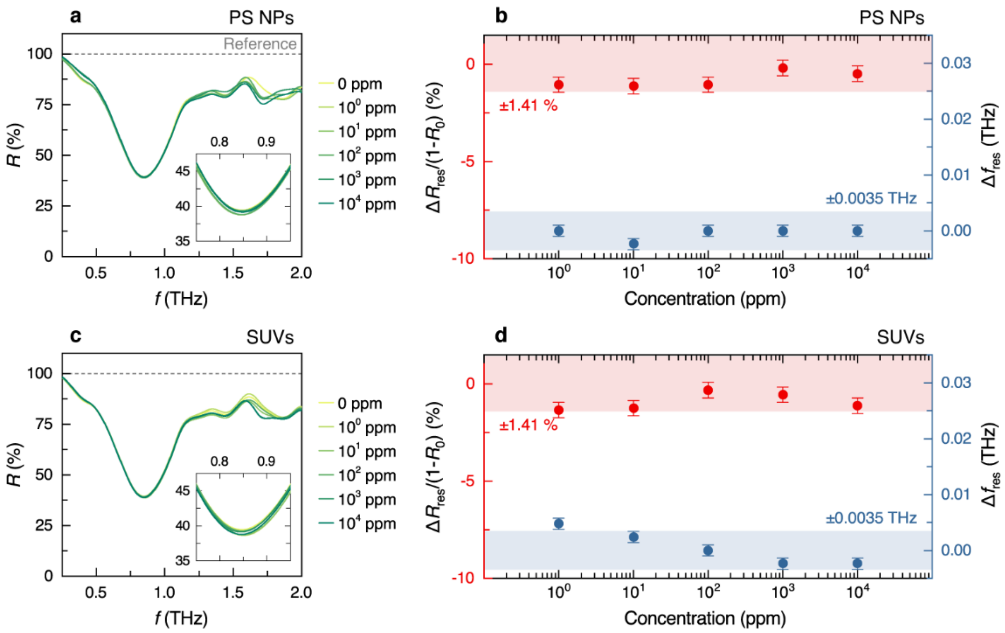

Figure S11. Control experiment II: THz signal detection without particle accumulation. a–**d**) Experimental time-lapse THz reflectance spectra (**a**, **c**) and time-lapse variation of $\Delta R_{res}/(1-R_0)$ and Δf_{res} (**b**, **d**) of nanoslot array measured using PS NP (**a**, **b**) and SUV solutions (**c**, **d**) under AC voltage of V_{pp} = 4 V and f = 1 kHz.

Supporting Note 7. Optical simulation of multi-phase accumulation of PS NPs by effective medium approximation.

Effective medium approximation for accumulation of PS NPs. To emulate the accumulation of NPs into the nanoslot region, an effective medium approximation based on MG theory was adopted by assuming NPs as inclusions and the aqueous environment as a host medium. Because a composite mixture of subwavelength-scaled inclusions in the host medium can be interpreted as a homogeneous system, the effective dielectric constant ($\varepsilon_{\mathrm{eff}}$) of the MG composite system is given as[30]

$$\varepsilon_{\mathrm{eff}} = \varepsilon_m \frac{2\varepsilon_m + \varepsilon_i + 2\delta_i(\varepsilon_i - \varepsilon_m)}{2\varepsilon_m + \varepsilon_i - \delta_i(\varepsilon_i - \varepsilon_m)}, \tag{S21}$$

where δ_i, ε_i, and ε_m are the volumetric fraction of inclusions, dielectric constants of inclusions, and host medium, respectively. Here, the maximum value of δ_{i} (δ_{max}) can be given as $\delta_{\mathrm{max}} = \pi/3\sqrt{2} \approx 74.0$ %, assuming that the structure of maximum nanoparticulate inclusions in the host medium approaches hexagonal close packing[31]. Thus, the volumetric fraction of nanoparticulate inclusions (δ_{i}) can be represented as $\delta_{\mathrm{i}} = \chi_{\mathrm{i}} \cdot \delta_{\mathrm{max}}$, by introducing a partial volumetric parameter of inclusions, χ_{i} ($0 < \chi_{\mathrm{i}} < 1$), as a parameter to indicate the degree of inclusion filling. Therefore, the volumetric filling fractions of PS NPs (δ_{PS}) can be represented as

$$\delta_{\mathrm{PS}} = \chi_{\mathrm{PS}} \cdot \delta_{\mathrm{max}}, \tag{S22}$$

where χ_{PS} denotes a partial volumetric parameter of PS NPs. Based on the optical properties of water and PS (**Figures S12a** and **S12b**) , the effective refractive index (n_{eff}) of MG and the extinction coefficient (κ_{eff}) were calculated using **Equations S21** and **S22** as a function of δ (**Figures S13a** and **S13b**).

Optical simulation of the multi-phase model. Based on the multi-stage accumulation process (**Main Figures 4b–e**), far-field THz reflectance was calculated by optical simulation with the geometric domains of the MG's effective optical properties adopted into the nanoslot region of the numerical model (**Supporting Note 2**). In this simulation model for multi-stage accumulation, a set of two rod-like geometries are assigned at T_1 sites along the longitudinal direction of the nanoslot, the y-axis. These rod-like geometries represent NP accumulation at T_1 sites with their optical properties as the effective MG values of PS NPs. To emulate the initial state of PS NP accumulation, stages I and II, reflections were calculated as the radius of rods increased considering the NPs pile up. When the top ends of the rods approached the T_2 sites, another set of two rod-like geometries was newly arranged at the S_2 sites to emulate the accumulation process at the T_2 sites. The reflection spectra at stages III and IV were calculated by increasing the radius of the newly added rods, while the previous set of rods remained

consistent. As evaluated from the far-field THz reflectance spectra collected above, $\Delta R_{res}/(1 - R_0)$ and Δf_{res} were plotted as a function of the geometric parameter v/v_{slot} and χ, where v and v_{slot} note for a volume of rod-like geometries and slot region, $v_{slot} = w(t_1 + t_2 + t_3)L$, respectively, exhibiting different slopes of curves as they passed through different stages of accumulation (**Figure S14a**). Then, the Michaelis-Menten function, defined as

$$v(t) = \frac{v_{max} t}{k_m + t}, \tag{S23}$$

was adopted to transform the geometric domain into the time domain with the assumption that the amount of NP accumulation increases over time and becomes saturated (estimated from **Main Figures 2e** and **2f**). For a 10 ppm PS NP solution, the Michaelis-Menten parameters of $v_{max} = 1.9v_{slot}$ and $k_m = 9.8$ were utilized, where v_{max} and k_m denote the maximum saturation and slope of the curve (**Figure S14b**).

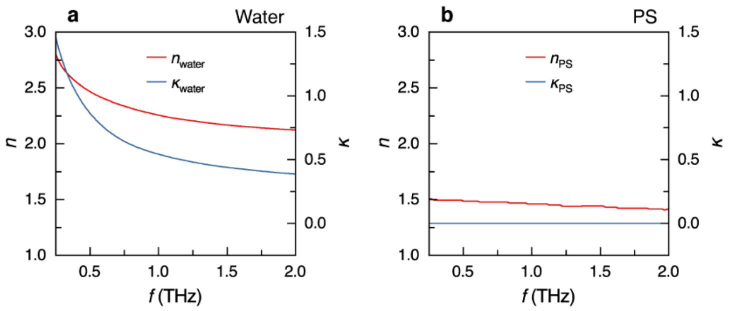

Figure S12. Optical properties of materials required for emulating PS NP accumulation. a–b) Refractive indices and extinction coefficients of water (**a**) and PS (**b**), obtained from references [3] and [4], respectively.

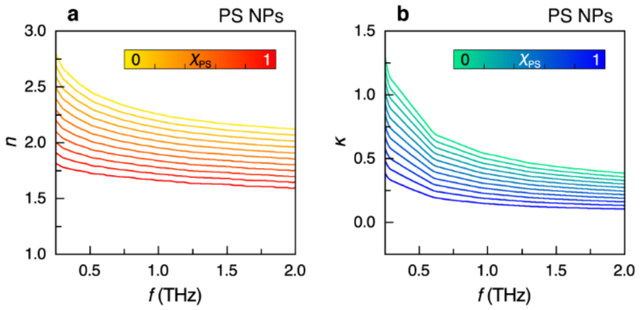

Figure S13. MG effective approximation for emulating PS NP accumulation. a–b) Effective MG refractive index (**a**) and extinction coefficient (**b**) of PS NPs in water host medium as function of χ_{PS}, from 0 to 1 every 0.1 steps.

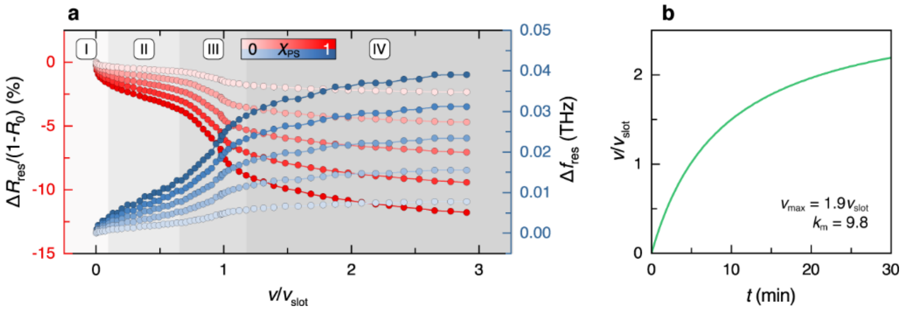

Figure S14. Optical simulation for a multi-stage PS NP accumulation. a) Simulational $\Delta R_{res}/(1\text{-}R_0)$ and Δf_{res} of multi-stage accumulation model of PS NPs as a function of v/v_{slot} and χ_{PS}, from 0.2 to 1 every 0.2 steps. **b**) Michaelis-Menten function for modelling time-lapse accumulation of 10 ppm PS NPs as function of t (v_{max} = 1.9v_{slot}, and k_m = 9.8).

Supporting Note 8. Simulation of a mono-stage accumulation of PS NPs.

To rationalize the multi-stage accumulation model described in the main manuscript, a mono-stage accumulation model consisting only of a single stage was adopted as a control for the multi-stage model. In this verification process, the rod-like geometries described above were assigned to constantly vary in only a single stage without any additional arrangement of rods, followed by the calculation of $\Delta R_{res}/(1 - R_0)$ and Δf_{res} with the same Michaelis-Menten function presented above (**Figure S14b**). First, assuming that the trapping characteristics of T_1 and T_2 are identical, a mono-stage model with simultaneous accumulations at four different sites of T_1 and T_2 were considered (**Figure S15a**). According to the simulation results as a function of geometric (**Figure S15b**) and time-dependent parameters (**Figure S15c**), both results exhibit rapid changes in the first step. Then, their gradual levelling off begins since trapping starts from the hotspot sites and accumulates further away from those sites as the process proceeds. Second, considering the previous study that showed that simultaneous interaction between DEP and ACEO might gather NPs into the centre of the pattern[32], a mono-stage model in which accumulation initiates from the centre inside the slot was presented (**Figure S16a**). As shown in the optical simulation of the geometric domain (**Figure S16b**), the slopes of the graph become steeper as they approach the sensing hotspots at the tips and show a gradual levelling off after occupying hotspot sites, although this trend is difficult to identify in the time domain (**Figure S16c**).

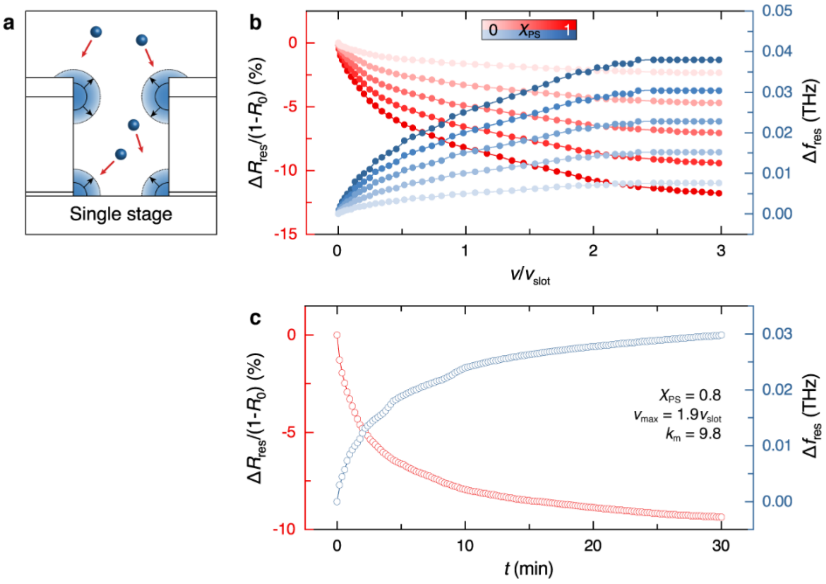

Figure S15. Optical simulation of mono-stage accumulation at nanoslot edges. a) Schematic of mono-stage accumulation at edges of nanoslot region. **b**) Simulational $\Delta R_{res}/(1\text{-}R_0)$ and Δf_{res} of mono-stage accumulation model of PS NPs as a function of v/v_{slot} and χ_{PS}, from 0.2 to 1 every 0.2 steps. **c**) Simulational $\Delta R_{res}/(1\text{-}R_0)$ and Δf_{res} of mono-stage accumulation model of PS NPs as a function of t using the Michaelis-Menten function (χ_{PS} = 0.8, v_{max} = 1.9v_{slot}, and k_m = 9.8).

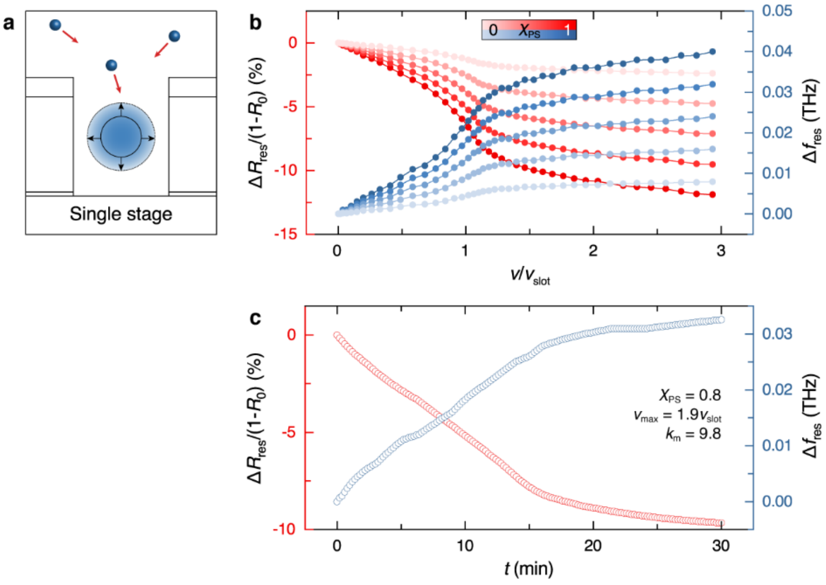

Figure S16. Optical simulation of mono-stage accumulation at nanoslot centre. a) Schematic of mono-stage accumulation at center of nanoslot region. **b**) Simulational $\Delta R_{res}/(1\text{-}R_0)$ and Δf_{res} of mono-stage accumulation model of PS NPs as function of v/v_{slot} and χ_{PS}, from 0.2 to 1 every 0.2 steps. **c**) Simulational $\Delta R_{res}/(1\text{-}R_0)$ and Δf_{res} of mono-stage accumulation model of PS NPs as function of t using the Michaelis-Menten function (χ_{PS} = 0.8, v_{max} = 1.9v_{slot}, and k_m = 9.8).

Supporting Note 9. Measuring optical properties of target materials.

Extracting optical properties from pelletized materials. To extract optical properties from target materials, the pelletization method and the Beer-Lambert formalism are commonly used[33]. After preparing the target materials into optically thick pellets, THz pulses were propagated through the pellets and reference (air) using a transmissive THz TDS system. Then, the time-domain transmitted electric fields of $E_{ref}(t)$ and $E_{sam}(t)$ were obtained and Fourier-transformed into frequency-domain spectra $E_{ref}(\omega)$ and $E_{sam}(\omega)$, where $E(\omega)$ can be separated into amplitude $A(\omega)$ and phase $\phi(\omega)$, $E(\omega) = A(\omega)e^{i\phi(\omega)}$. In the case of optically thick pellets, the relation between $E_{sam}(\omega)$ and $E_{ref}(\omega)$ can be expressed with complex refractive indices of the target material ($\tilde{n}_{sam}$) and reference ($\tilde{n}_{ref}$) as follows[34]:

$$\frac{E_{sam}(\omega)}{E_{ref}(\omega)} = \exp\left[i \;\; \tilde{n}_{sam} - \tilde{n}_{ref} \;\; \frac{\omega d}{c} \right], \tag{S24}$$

where d and c denote the thickness of a pellet sample and the speed of light, respectively. Because these complex refractive indices can be expressed as $\tilde{n}_{ref} = 1$ (air as reference) and $\tilde{n}_{sam} = n(\omega) + i\kappa(\omega)$, where $n(\omega)$ and $\kappa(\omega)$ are the refractive index and extinction coefficient of the sample material, **Equation S24** can be represented as

$$\frac{E_{sam}(\omega)}{E_{ref}(\omega)} = \exp\left\{ -\frac{d\alpha(\omega)}{2} \right\} \cdot \exp\left[i \;\; n(\omega) - 1 \;\; \frac{2\pi d}{\lambda} \right], \tag{S25}$$

where λ is the wavelength and α is the absorption coefficient ($\alpha = 4\pi\kappa/\lambda$). Therefore, the $n(\omega)$ and $\alpha(\omega)$ of materials can be extracted from the difference in the phase and spectral amplitude between the transmitted signal as follows:

$$n(\omega) = 1 + \frac{\lambda}{2\pi d}\Delta\phi(\omega) \text{ and } \alpha(\omega) = -\frac{1}{d}\ln \;\; T(\omega) \;\; , \tag{S26}$$

where $T(\omega)$ and $\Delta\phi(\omega)$ are the transmittance and phase difference between two waveform signals, $T(\omega) = |E_{sam}(\omega)/E_{ref}(\omega)|^2$ and $\Delta\phi(\omega) = \phi_{sam}(\omega) - \phi_{ref}(\omega)$, respectively.

Effective medium approximation for heterogeneous pelletization. In contrast to mechanically stable materials that can be easily assembled into optically thick homogeneous pellets, some materials show pelletization difficulty owing to mechanical instability. As an alternative, the target materials are assembled into heterogeneous mixture pellets along with a filling material such as polyethylene (PE). While optical properties can be extracted directly from homogeneous pellets, extracting the optical properties of the target material from mixture pellets requires effective medium approximation by MG theory[30]. Because a composite mixture with subwavelength-scaled inclusions within the host medium can be interpreted as a homogeneous system, the effective dielectric constant of the mixture (ε_{eff}) follows **Equation S21**. After optically measuring the dielectric constants of PE (ε_{PE}) and mixture, the optical properties of the target material (ε_i) were derived from **Equation S21** with the assumption of

modelling mixture pellets as an MG effective medium where spherical target materials are inclusions embedded within the PE filler matrix ($\varepsilon_m = \varepsilon_{\mathrm{PE}}$). In this process, the value of δ_i, which represents the volumetric fraction of target inclusions in the medium, was evaluated from

$$\delta_i = \frac{m_i / \rho_i}{(m_i / \rho_i) + (m_{\mathrm{PE}} / \rho_{\mathrm{PE}})}, \tag{S27}$$

where m_i, m_{PE}, ρ_i, and ρ_{PE} are the mass and densities of the target material and PE filler, respectively.

Pelletization and measurement of target materials. Generally, optically thick pellets of target materials are prepared by pressing powdered materials. However, the pressing technique cannot be applied to achieve a PVP/PMF pellet because the cross-linking agent of PMF is a liquid. Therefore, as a prior step for preparing a PVP/PMF pellet, a 5 mm thick large poly(dimethylsiloxane) (PDMS) corral was prepared by curing the PDMS mixture (elastomer:curing agent = 10:1, Sylgard 184, Dow Corning) on a hotplate at 80 °C overnight. Using a plasma system (Cute plasma system, Femto Science), O_2 plasma treatment (O_2 gas flow rate of 30 sccm, RF power of 70 W, pressure of 0.5 Torr, and duration time of 40 s) was carried out on the surface of the PDMS corral and a Si wafer, followed by a subsequent attachment of PDMS on the Si wafer. After assembly, the PVP/PMF mixture solution was poured into the PDMS corral and cured by annealing at 100 °C overnight and post-annealing at 200 °C for 20 min. Then, the cured PVP/PMF with a thickness of 620 μm was detached from the wafer and ground into an 8 mm diameter round pellet.

Owing to the insufficient mechanical stability of the DOPC pellet, DOPC powder (850375, Avanti Polar Lipids) was mixed with a larger amount of filler material powder, high-density PE (Sigma Aldrich), and arranged into a mixture pellet[35]. After grinding powders of PE and DOPC for 15 min using a mortar and a pestle, the PE powder (16 mg) and mixture powder of DOPC/PE with a weight ratio of 1:2.23 (32 mg) were prepared and placed into a (1/4)" pellet pressing die (EQ-Die-06D-B, MTI). Subsequently, DOPC/PE and PE powders in a pellet pressing die were pressed under a pressure of 300 bar for 15 min using a pellet press (Jewelpes SMPG10, Smart-Hydraulic). Consequently, 8 mm diameter pellets of PE and DOPC/PE were prepared with thicknesses of 601 and 994 μm, respectively.

After completing pelletization (**Figure S17a**), the optical properties of the target materials were extracted by employing a THz TDS system in the transmission mode-. In this measurement process, the THz TDS system in the transmission mode was arranged by replacing several modules from the reflection mode. For transmission measurements, a THz pulse generated from the photoconductive antenna was transmitted through a sample stage and focused on a ZnTe detector, where the waves were collimated and focused onto the sample stage by a

polymethylpentene lens (TPX). As a consequence, the $n(\omega)$ and $\alpha(\omega)$ of the target materials, including PVP/PMF, PE, and DOPC/PE, were obtained in the spectral range of 0.2 to 2.0 THz (**Figures S17b** and **S17c**). During the entire process, the signals were measured 30 times and averaged to obtain stable optical signals.

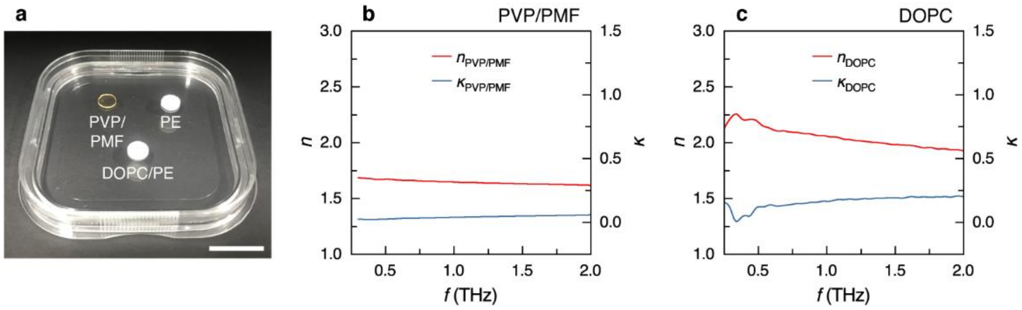

Figure S17. Optical properties measured from pelletized target materials. a) Photograph of PVP/PMF (upper-left), PE (upper-right), and DOPC/PE pellets (lower). Scale bar, 1 cm. **b**–**c**) Refractive indices and extinction coefficients of PVP/PMF (**b**) and DOPC (**c**), obtained from measurement.

Supporting Note 10. Simulation of multi-phase accumulation of SUVs by effective medium approximation.

Effective medium approximation for the accumulation of SUVs. In contrast to the homogeneous PS NPs, which are full of PS materials, SUVs consist of a heterogeneous structure of a surrounding lipid bilayer membrane and an inner medium. Therefore, the volume of DOPC in a single SUV (v_{DOPC}) can be given by the difference between those of the SUV (v_{SUV}) and the inner medium (v_{in}); $v_{DOPC} = v_{SUV} - v_{in} = (4/3)\pi\{r^3 - (r - d_{mem})^3\}$, where r and d_{mem} denote the diameter and membrane thickness of the SUV. For SUVs with a diameter of 50 nm (r = 25 nm and d_{mem} = 5 nm[36]), the volumetric ratio of DOPC in a single SUV (v_{DOPC}/v_{SUV}) was estimated to be 48.8 %, while that of the inner medium (v_{in}/v_{SUV}) accounted for 51.2 %. Considering that SUV accumulation follows the effective medium approach of the MG ($\delta_{SUV} = \chi_{SUV}\cdot\delta_{max}$, where δ_{SUV} and χ_{SUV} denote a volumetric fraction and a partial volumetric parameter of SUVs), the volumetric filling fraction of DOPC in heterogeneous SUVs (δ_{DOPC}) can be expressed as

$$\delta_{DOPC} = (v_{DOPC}/v_{SUV})\cdot\delta_{SUV} = \chi_{SUV}\cdot[1 - \{1 - (d_{mem}/r)\}^3]\cdot\delta_{max}. \qquad \text{(S28)}$$

By utilizing the optical properties of the materials (**Figures S12a** and **S17c**) and **Equation S21**, the effective optical constants of MG in the water medium were obtained as a function of χ_{SUV} (**Figures S18a** and **S18b**). Then, $\Delta R_{res}/(1 - R_0)$ and Δf_{res} of SUVs as a function of v/v_0 were calculated by optical simulation based on the multi-stage accumulation described above (**Supporting Note 7**), exhibiting reduced shifts compared with those of PS NPs (**Figure S14a**). Finally, considering that the solution concentration of SUVs (1 ppm) is much lower than that of PS NPs (10 ppm), the Michaelis-Menten function for slower accumulation over time was adopted with their parameters, $v_{max} = 2.8v_{slot}$ and $k_m = 40$ (**Figure S14b** and **Main Figure 4**).

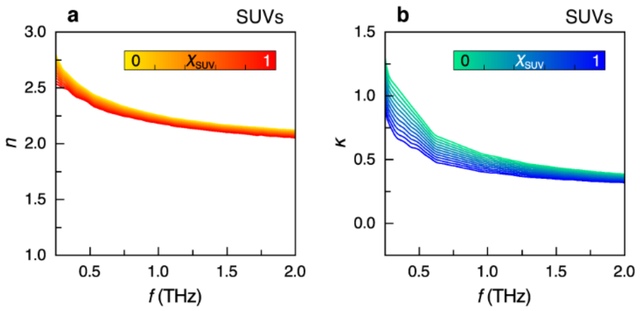

Figure S18. MG effective approximation for emulating SUV accumulation. a–b) Effective refractive index of MG (**a**) and extinction coefficient (**b**) of SUVs in water host medium as a function of χ_{SUV}, from 0 to 1 every 0.1 steps.

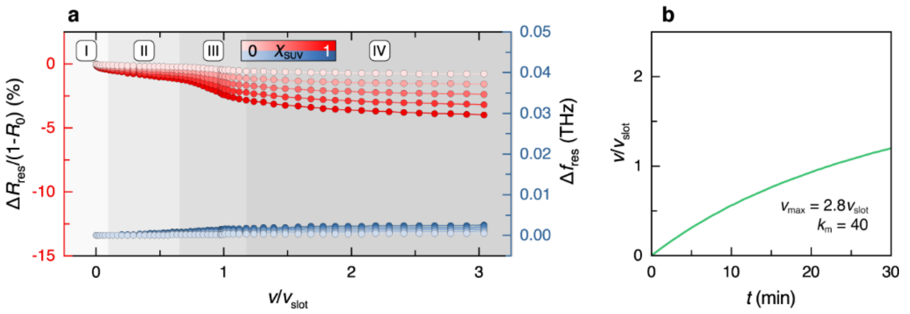

Figure S19. Optical simulation for multi-stage SUV accumulation. a) Simulational $\Delta R_{res}/(1-R_0)$ and Δf_{res} of multi-stage accumulation model of SUVs as a function of v/v_{slot} and χ_{SUV}, from 0.2 to 1 every 0.2 steps. **b**) the Michaelis-Menten function for modelling time-lapse accumulation of 1 ppm SUVs as a function of t ($v_{max} = 2.8v_{slot}$ and $k_m = 40$).

Supporting Movies

Movie M1. Image-processed FL micrograph videos (upper) and corresponding FL plot-profile (lower) on nanoslot array with solutions of PS NPs under various V_{pp} values and time-lapse sweeping of f while switching on and off.

Movie M2. Image-processed FL micrograph videos (upper) and corresponding FL plot-profile (lower) on nanoslot array with solutions of SUVs under various V_{pp} values and time-lapse sweeping of f while switching on and off.

Supporting References

[1] H.-R. Park, K. J. Ahn, S. Han, Y.-M. Bahk, N. Park, D.-S. Kim, *Nano Lett.* **2013**, 13, 1782.

[2] D. Grischkowsky, S. Keiding, M. van Exter, C. Fattinger, *J. Opt. Soc. Am. B* **1990**, 7, 2006.

[3] J. E. Bertie, Z. Lan, *Appl. Spectrosc.* **1996**, 50, 1047.

[4] M. Naftaly, R. E. Miles, P. J. Greenslade, "THz transmission in polymer materials — a data library", presented at *2007 Joint 32nd International Conference on Infrared and Millimeter Waves and the 15th International Conference on Terahertz Electronics*, 2-9 Sept. 2007, 2007.

[5] H. Morgan, N. G. Green, *AC Electrokinetics: Colloids and Nanoparticles*, Research Studies Press, Baldock **2003**.

[6] A. Castellanos, A. Ramos, A. Gonzalez, N. G. Green, H. Morgan, *J. Phys. D: Appl. Phys.* **2003**, 36, 2584.

[7] O. Stern, *Z. Elktrochem. Angew. Phys. Chem.* **1924**, 30, 508.

[8] N. G. Green, A. Ramos, A. González, H. Morgan, A. Castellanos, *Phys. Rev. E* **2002**, 66, 026305.

[9] A. González, A. Ramos, N. G. Green, A. Castellanos, H. Morgan, *Phys. Rev. E* **2000**, 61, 4019.

[10] H. V. Helmholtz, *Ann. Phys.* **1879**, 243, 337.

[11] J. Oh, R. Hart, J. Capurro, H. M. Noh, *Lab Chip* **2009**, 9, 62.

[12] A. Ramos, H. Morgan, N. G. Green, A. Castellanos, *J. Phys. D: Appl. Phys.* **1998**, 31, 2338.

[13] D. Chen, H. Du, *J. Micromech. Microeng.* **2006**, 16, 2411.

[14] D. R. Lide, *CRC Handbook of Chemistry and Physics*, CRC Press, Boca Raton **2012**.

[15] B. R. Munson, *Fundamentals of Fluid Mechanics*, John Wiley & Sons, Hoboken **2013**.

[16] M. P. N. Juniper, A. V. Straube, D. G. A. L. Aarts, R. P. A. Dullens, *Phys. Rev. E* **2016**, 93, 012608.

[17] D. P. Fernández, Y. Mulev, A. R. H. Goodwin, J. M. H. L. Sengers, *J. Phys. Chem. Ref. Data* **1995**, 24, 33.

[18] J. Henninges, E. Huenges, H. Burkhardt, *J. Geophys. Res.: Solid Earth* **2005**, 110, B11206.

[19] T. M. Tritt, *Thermal Conductivity: Theory, Properties, and Applications*, Kluwer Academic/Plenum Publishers, New York **2004**.

[20] K. J. Baeg, Y. Y. Noh, J. Ghim, B. Lim, D. Y. Kim, *Adv. Funct. Mater.* **2008**, 18, 3678.

[21] Y.-W. Kwon, C. H. Lee, D.-H. Choi, J.-I. Jin, *J. Mater. Chem.* **2009**, 19, 1353.

[22] G.-H. Kim, D. Lee, A. Shanker, L. Shao, M. S. Kwon, D. Gidley, J. Kim, K. P. Pipe, *Nat. Mater.* **2015**, 14, 295.

[23] S. N. Sze, *Physics of Semiconductor Devices*, John Wiley, New York **1981**.

[24] C. D. Thurmond, *J. Electrochem. Soc.* **1975**, 122, 1133.

[25] W. W. Gartner, *Proc. IRE* **1957**, 45, 662.

[26] C. J. Glassbrenner, G. A. Slack, *Phys. Rev.* **1964**, 134, A1058.

[27] E. Herth, S. Seok, N. Rolland, T. Lasri, *Sensor. Actuat. A: Phys.* **2012**, 173, 238.

[28] R. A. Serway, J. W. Jewett, *Principles of Physics*, Saunders College Publishing, Fort Worth **1998**.

[29] J. Kestin, M. Sokolov, W. A. Wakeham, *J. Phys. Chem. Ref. Data* **1978**, 7, 941.

[30] J. C. M. Garnett, J. Larmor, *Philos. Trans. Royal Soc. A* **1904**, 203, 385.

[31] J. H. Conway, N. J. A. Sloane, *Sphere Packings, Lattices and Groups*, Springer, New York **2013**.

[32] E.-S. Yu, H. Lee, S.-M. Lee, J. Kim, T. Kim, J. Lee, C. Kim, M. Seo, J. H. Kim, Y. T. Byun, S.-C. Park, S.-Y. Lee, S.-D. Lee, Y.-S. Ryu, *Nat. Commun.* **2020**, 11, 2804.

[33] L. Duvillaret, F. Garet, J.-L. Coutaz, *IEEE J. Sel. Top. Quant.* **1996**, 2, 739.

[34] M. Seo, H.-R. Park, *Adv. Opt. Mater.* **2020**, 8, 1900662.

[35] E. P. Parrott, J. A. Zeitler, L. F. Gladden, *Opt. Lett.* **2009**, 34, 3722.

[36] a) T.-Y. Yoon, C. Jeong, S.-W. Lee, J. H. Kim, M. C. Choi, S.-J. Kim, M. W. Kim, S.-D. Lee, *Nat. Mater.* **2006**, 5, 281; b) Y.-S. Ryu, I.-H. Lee, J.-H. Suh, S. C. Park, S. Oh, L. R. Jordan, N. J. Wittenberg, S.-H. Oh, N. L. Jeon, B. Lee, A. N. Parikh, S.-D. Lee, *Nat. Commun.* **2014**, 5, 4507; c) Y.-S. Ryu, D. Yoo, N. J. Wittenberg, L. R. Jordan, S.-D. Lee, A. N. Parikh, S.-H. Oh, *J. Am. Chem. Soc.* **2015**, 137, 8692; d) Y.-S. Ryu, L. R. Jordan, N. J. Wittenberg, S. M. Kim, D. Yoo, C. Jeong, A. E. Warrington, M. Rodriguez, S.-H. Oh, S.-D. Lee, *Adv. Mater. Interfaces* **2018**, 5, 1801290; e) Y.-S. Ryu, H. Yun, T. Chung, J.-H. Suh, S. Kim, K. Lee, N. J. Wittenberg, S.-H. Oh, B. Lee, S.-D. Lee, *Biosens. Bioelectron.* **2019**, 142, 111568.
